# Estimating yield-contributing physiological parameters of cotton using UAV-based imagery

**DOI:** 10.3389/fpls.2023.1248152

**Published:** 2023-09-19

**Authors:** Amrit Pokhrel, Simerjeet Virk, John L. Snider, George Vellidis, Lavesta C. Hand, Henry Y. Sintim, Ved Parkash, Devendra P. Chalise, Joshua M. Lee, Coleman Byers

**Affiliations:** ^1^ Department of Crop and Soil Sciences, University of Georgia, Tifton, GA, United States; ^2^ College of Engineering, University of Georgia, Athens, GA, United States

**Keywords:** fraction of intercepted photosynthetically active radiation, radiation use efficiency, harvest index, unmanned aerial vehicles, RGB imagery, multispectral imagery

## Abstract

Lint yield in cotton is governed by light intercepted by the canopy (IPAR), radiation use efficiency (RUE), and harvest index (HI). However, the conventional methods of measuring these yield-governing physiological parameters are labor-intensive, time-consuming and requires destructive sampling. This study aimed to explore the use of low-cost and high-resolution UAV-based RGB and multispectral imagery 1) to estimate fraction of IPAR (IPAR_f_), RUE, and biomass throughout the season, 2) to estimate lint yield using the cotton fiber index (CFI), and 3) to determine the potential use of biomass and lint yield models for estimating cotton HI. An experiment was conducted during the 2021 and 2022 growing seasons in Tifton, Georgia, USA in randomized complete block design with five different nitrogen treatments. Different nitrogen treatments were applied to generate substantial variability in canopy development and yield. UAV imagery was collected bi-weekly along with light interception and biomass measurements throughout the season, and 20 different vegetation indices (VIs) were computed from the imagery. Generalized linear regression was performed to develop models using VIs and growing degree days (GDDs). The IPAR_f_ models had R^2^ values ranging from 0.66 to 0.90, and models based on RVI and RECI explained the highest variation (93%) in IPAR_f_ during cross-validation. Similarly, cotton above-ground biomass was best estimated by models from MSAVI and OSAVI. Estimation of RUE using actual biomass measurement and RVI-based IPAR_f_ model was able to explain 84% of variation in RUE. CFI from UAV-based RGB imagery had strong relationship (R^2 ^= 0.69) with machine harvested lint yield. The estimated HI from CFI-based lint yield and MSAVI-based biomass models was able to explain 40 to 49% of variation in measured HI for the 2022 growing season. The models developed to estimate the yield-contributing physiological parameters in cotton showed low to strong performance, with IPAR_f_ and above-ground biomass having greater prediction accuracy. Future studies on accurate estimation of lint yield is suggested for precise cotton HI prediction. This study is the first attempt of its kind and the results can be used to expand and improve research on predicting functional yield drivers of cotton.

## 1 Introduction

Cotton (*Gossypium* sp.) is one of the most important crops to the global textile and clothing industry (Sui et al., 2017; Lu, 2022). Cotton production supports the trillion dollar fashion industry, attracts revenues for countries, and provides employment opportunities to millions of people (Voora et al., 2020). The United Sates is a prime producer as well as exporter of this natural fiber, producing around 3 MT of cotton ($6 billion worth of value) and providing around 30% of global exports (USDA, 2022; USDA, 2023). Because of its significance, the majority of breeding and crop management efforts in the United States and Australia have been focused on lint yield improvements and yield stability (Conaty and Constable, 2020; Snider et al., 2021b). Physiologically, yield is a function of the cumulative amount of photosynthetically active radiation intercepted by the canopy during the growing season (IPAR), the efficiency with which a crop converts intercepted radiation into biomass (RUE), and the fraction of total biomass allocated to the economically important part of the crop, or harvest index (HI) (Monteith, 1972). Thus, yield improvements or declines resulting from breeding or management efforts can be attributed to alterations in any or all of the aforementioned traits. Nitrogen is one of the most important yield-governing factors that influences cotton growth and development. For example, nitrogen deficiency decreases leaf area expansion and canopy development (Wullschleger and Oosterhuis, 1990), which could reduce IPAR (Wajid et al., 2010). Reductions in single-leaf and whole-canopy photosynthetic rates commonly occur under nitrogen deficiency (Bondada and Oosterhuis, 2001; Snider et al., 2021a; Parkash et al., 2023), and previous reports have documented positive associations between N application rates and whole-canopy RUE (Bange and Milroy, 2000). Due to a reduction in the ability of the canopy to support fruit development under N deficiency or due to excessive vegetative growth and low fruit retention under N excess, yield can be significantly reduced (Gerik et al., 1994). As a result, it is important to understand the response of underlying physiological parameters (IPAR, RUE, and HI) to yield-altering N application rates. Traditionally in small-plot research, IPAR is calculated by measuring above and below canopy light intensity, RUE estimation requires above ground dry weight samples, and HI measurement needs hand harvested samples of cotton. However, these traditional methods of measuring the physiological parameters are both time and labor intensive, and require destructive sampling of plants (Ermanis et al., 2020).

Due to most physiological measurements being laborious and time-consuming, remote sensing has the potential of becoming a rapid and efficient non-destructive method for characterizing crop and vegetation bio-physical properties (Wiegand et al., 1991; Kumar et al., 2002). Over the years, remote sensing platforms have evolved from low-resolution orbital satellites in the 1970s to advanced unmanned aerial vehicles (UAVs) in recent years. In comparison to satellites, UAVs provide many improved features such as stability, reliability, autonomy, affordability, flights at lower altitudes, optimum data quality, high spatial and temporal resolution (Sankaran et al., 2015; Barbedo, 2019). While UAVs also have some limitations such as short battery life, technical knowledge, and limited airspace authorization (Jafarbiglu and Pourreza, 2022) they still provide an opportunity for rapid, reliable, and non-destructive measurement of crop biophysical characteristics. Furthermore, the availability of reliable and high spectral resolution sensors, such as Red Green Blue (RGB) (Bendig et al., 2015), multispectral (mainly red, blue, green, red-edge, and near-infrared spectral bands) (Deng et al., 2018), hyperspectral (a wide range of spectral bands) (Zhang et al., 2018), thermal (Gonzalez-Dugo et al., 2013), and depth (LiDAR) (Sun et al., 2018) have greatly expanded the capabilities of UAVs in agriculture. Low-cost sensors such as RGB and multispectral sensors on small, light-weight UAVs are commonly used by agricultural researchers because of their dependability, affordability, and ease of image processing and analysis (Jafarbiglu and Pourreza, 2022). RGB imagery has primarily been used for extracting soil or crop characteristics of interest (Huang et al., 2016; Li et al., 2019), and estimating crop heights (Bendig et al., 2015). By comparison, multispectral imagery incorporates red-edge (670-760 nm) and near-infrared (760-900 nm) wavebands that are inaccessible when using traditional RGB imagery (Ashapure et al., 2019). As a result, multispectral imagery has been widely used to generate vegetation indices (VIs) in many crops, which are used to estimate canopy cover (Xu et al., 2019), biomass (Yue et al., 2019; Wang et al., 2021), leaf area index (Boegh et al., 2002; Gutierrez et al., 2012), chlorophyll content (Raper and Varco, 2014), evapotranspiration (Hunsaker et al., 2003), nutrient status (Zhao et al., 2005; Ballester et al., 2017), and yield (Zhao et al., 2007b; Vatter et al., 2021).

Currently, limited studies have attempted to correlate VIs derived from UAV-based multispectral imagery to IPAR in cotton. However, VIs derived from satellite imagery, ground spectro-radiometers, and hyperspectral sensors have been shown as a suitable proxy for the fraction of incident PAR (IPAR_f_) intercepted by the canopy. For example, multiple studies have reported the Normalized Difference Vegetation Index (NDVI) to be a good predictor of IPAR_f_ for cotton (Prasad et al., 2021), garlic (Campoy et al., 2019), wheat (Tan et al., 2018; Pellegrini et al., 2020; Lv et al., 2021), corn (Tan et al., 2013), and soybean (Hatfield and Prueger, 2010). Furthermore, IPAR_f_ is highly dependent on crop canopy structure and architectural traits like leaf area index (LAI), and several studies have shown that the Ratio Vegetation Index (RVI), Green Normalized Difference Vegetation Index (GNDVI), Enhanced Vegetation Index (EVI) and NDVI can be used to predict cotton LAI (Zhao et al., 2007a; Gutierrez et al., 2012; Chen, 2019) with a moderate to high accuracy (R^2 ^= 0.70 to 0.93).

Reports on the relationship between multispectral VIs and RUE are also limited in cotton. Most studies on RUE estimation for leaf and canopy have used Photochemical Reflectance Index (PRI) and Sun-Induced Fluorescence (SIF) estimates, obtained from narrow-band hyperspectral reflectance data and fine resolution spectrometers, respectively (Garbulsky et al., 2011; Zhang et al., 2016; Zhang et al., 2018; Merrick et al., 2020; Fu et al., 2022). PRI uses reflectance data obtained from 531 and 570 nm wavelengths that measures changes in xanthophyll cycle and pigment ratios (chlorophyll/carotenoid), (Hilker et al., 2008; Garbulsky et al., 2011; Zhang et al., 2016), while SIF estimates the fluorescence emission in the far-red region (650-850 nm) from excited chlorophyll (Zhang et al., 2018; Merrick et al., 2020; Porcar-Castell et al., 2021), both of them ultimately relating to photosynthetic efficiency and RUE at leaf and canopy levels. For example, recent studies have used PRI or SIF or both to estimate RUE in crops like corn (Cheng et al., 2013) and wheat at the canopy level (Robles-zazueta et al., 2021), and cotton at the leaf level under water stress conditions (Zhang et al., 2018). However, obtaining SIF and hyperspectral VIs remains a challenge due to its high cost instruments, sensitivity to noise, and complex analytical procedures (Hilker et al., 2008; Porcar-Castell et al., 2021). Because chlorophyll content per unit leaf area can influence the quantum efficiency of primary photochemistry (Porcar-Castell et al., 2021), it is possible that multispectral VIs related to chlorophyll content are predictive of canopy-level RUE in cotton. Few studies have investigated the potential of multispectral VIs to estimate chlorophyll content, where Red-edge Chlorophyll Index (RECI) and Simplified Canopy Chlorophyll Content Index (SCCCI) are shown to be related to chlorophyll content in cotton and other crops (Raper and Varco, 2014; Ballester et al., 2017; Barbedo, 2019; Wang et al., 2021). Furthermore, previous research has shown that certain multispectral VIs, including NDVI, RVI, NIR, Normalized Difference Red-edge Index (NDRE), Modified Soil Adjusted Vegetation Index (MSAVI), Wide Dynamic Range Vegetation Index (WDRVI) can be used to estimate cotton biomass (Zhao et al., 2007a; Hatfield and Prueger, 2010; Gutierrez et al., 2012; Brandão et al., 2015; Chen and Wang, 2020). Because biomass is the product of IPAR (IPAR_f_ × PAR) and RUE (Conaty and Constable, 2020), the estimation of IPAR_f_ and biomass using multispectral VIs could potentially be used to derive RUE.

While the studies mentioned above have linked multispectral VIs to cotton LAI, biomass, and chlorophyll content, most of these relationships have been developed for a specific point in time or at a specific growth stage in these studies. As a result, the previously developed relationships are only predictive of these characteristics when measured at that specific time or growth stage. Reports have indicated that the relationship between VIs and crop growth characteristics such as LAI and biomass change with the phenological stage of the crop (Pinter et al., 2003; Gutierrez et al., 2012; Li Z. et al., 2022). A recent study from Li Z. et al. (2022) demonstrated significant improvements (R^2 ^= 0.83) in estimating the above-ground biomass of wheat throughout the season using multispectral VIs when used in conjunction with a well-established crop staging system such as growing degree days (GDD) or heat units. For cotton, crop growth and development are strongly tied to GDD accumulation (Ritchie et al., 2004; Sharma et al., 2021). Consequently, integrating GDD into VI-based functions can also be potentially used for predicting canopy-specific, yield-driving traits for cotton at any time during the season.

As crop biomass and yield can be predicted from aerial imagery (Gutierrez et al., 2012; Huang et al., 2016), it is likely that HI can be predicted from VIs used to derive these two traits. However, the authors are not aware of studies that have used multispectral imagery to capture in-field variation in HI for field-grown cotton. As discussed earlier, multiple VIs derived from multispectral imagery have shown a strong relationship with cotton biomass. Similarly, several studies have also suggested that multispectral imagery-based VIs such as NDVI, SCCI, NDRE and RVI as well as the individual reflectance bands including red and red-edge collected during the growing season have the potential to explain variation in lint yield (Yang et al., 2001; Zarco-Tejada et al., 2005; Zhao et al., 2007b; Gutierrez et al., 2012; Huang et al., 2013; Ballester et al., 2017). However, results from these studies indicate some discrepancies in the appropriate growth stage at which these VIs are effective, with few studies suggesting the relationship was better at the early flowering stage and others at the peak bloom stage. Huang et al. (2016) developed an alternate method to estimate cotton lint yield (R^2 ^= 0.83) after defoliation and before harvesting, based on the detection of white cotton pixels in an aerial RGB imagery. Feng et al. (2020) later named this ratio as Cotton Fiber Index (CFI) and showed a similar performance of CFI (R^2 ^= 0.90) in estimating cotton lint yield. CFI is the ratio of the number of white pixels (cotton bolls) to the total number of pixels in a given area and can be obtained from UAV-based RGB imagery following some image enhancement procedures.

Based on the available literature on the use of VIs to predict crop biophysical characteristics, VIs derived from UAV-based aerial imagery can potentially be utilized to predict yield-governing physiological parameters in field-grown cotton. However, studies predicting IPAR_f_, RUE, and HI for field-grown cotton using UAV-based RGB and/or multispectral imagery have not been published. Thus, the main objectives of our study were to: 1) develop and validate models to estimate IPAR_f_, RUE, and biomass of cotton throughout the season using VIs derived from UAV-based multispectral imagery and GDDs; 2) estimate cotton lint yield using cotton fiber index (CFI) extracted from UAV-based RGB imagery; and 3) investigate the potential of using biomass and lint yield estimates obtained from UAV-based RGB and multispectral imagery, respectively, to estimate cotton HI.

## 2 Materials and methods

### 2.1 Study site details and experimental design

**Figure 1:**
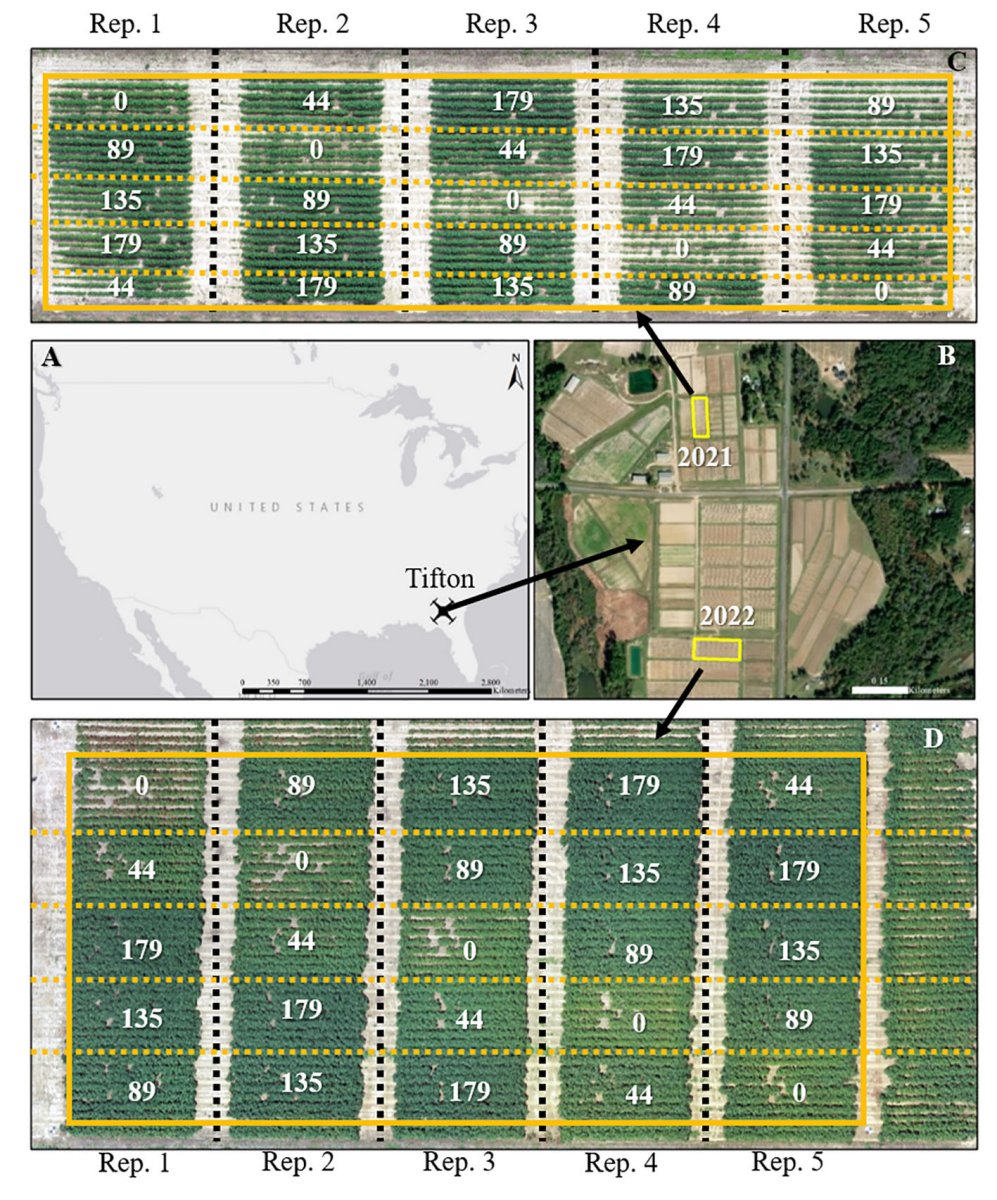
Geographical location of the study sites for the 2021 and 2022 growing seasons **(A, B)**. Aerial imagery of the field sites and layout of the experimental plots during the 2021 **(C)** and 2022 **(C)** growing seasons. A solid yellow box depicts the experimental area; black dashed lines separate replications, and yellow dashed lines separate each plot. Values in **(C, D)** are the nitrogen application rates in kg N ha^-1^.

The study was conducted at the Lang-Rigdon Farm (**Figure 1**) of the University of Georgia Tifton Campus in Tifton, Georgia, USA (31° 52’ N, 83° 55’ W) where the predominant soil type is classified as Tifton loamy sand (fine-loamy, kaolinite, plinthic kandiudults) (Soil Survey Staff, Natural Resources Conservation Service and United States Department of Agriculture, no date). During the 2021 and 2022 growing seasons, a field experiment was arranged in a randomized complete block design with five replications using a cotton cultivar DP 1646 B2XF and five nitrogen application rates of 0, 44, 89, 135, and 179 kg N ha^-1^. DP 1646 B2XF was the most widely grown cotton cultivar in the southeastern US during the time of this experiment (USDA Agricultural Marketing Service, 2020), and the five nitrogen application rates were implemented to create variability in crop growth and yield. N was applied as granular urea (46-0-0): 25% at planting and 75% at the initiation of floral bud development (squaring stage). All other agronomic management practices, except N application, were conducted based on the recommendations outlined in the University of Georgia Cotton Production Guide (Hand et al., 2022). The cotton crop was sown on June 1, 2021 and April 26, 2022, and machine harvested on October 25, 2021 and September 22, 2022. The weather data which includes daily maximum and minimum temperatures, and daily solar radiation (**Supplementary Figure 1**) for the study site from planting until harvest was obtained from the University of Georgia Weather Network (http://www.georgiaweather.net/). The 2022 growing season had higher average daily minimum and maximum temperatures by 0.15°C and 1.16°C, respectively than the 2021 growing season. The 2021 growing season had cloudier days with daily solar radiation below 10 MJ m^-2^ than the 2022 growing season. Daily maximum and minimum temperatures were used to calculate GDDs throughout the season for cotton during both years.

The accumulated GDDs of cotton over time is a predictor of growth and phenological stage during the season. The GDDs is calculated based on the average daily temperatures and base temperature of cotton using equation 1 (Ritchie et al., 2004).


(1) $$\text{GDDs}=\sum_{i=1}^{n}\left[\frac{\text{ Max. Temperature }{}^{\circ}{\text{C}}_{i}+\text{Min. Temperature }{}^{\circ}{\text{C}}_{i}}{2}-\text{Base Temperature }{}^{\circ}\text{C}\right]\;$$

where *i =1* signifies first day of planting and *n* is each sampling date. For cotton, the base temperature is 15.6°C (Snider et al., 2021b; Hand et al., 2022) and the upper threshold of maximum temperature is 33.9°C (Hand et al., 2022). The growth and development of cotton plants is assumed to be zero below the base temperature and above the upper threshold temperature.

### 2.2 Data collection

Physiological measurements and UAV-based imagery were collected on the same dates, beginning 5 weeks after planting, throughout the 2021 and 2022 growing seasons in approximately two-week intervals, depending on weather conditions on target sampling dates. The actual dates of data collection along with the GDD for each sampling date are provided in **Table 1**.

**Table 1:** Information on sampling date and types of measurements taken during the 2021 and 2022 growing seasons.

Year	DAP	Date	GDDs	Measurements	UAV-based imagery
2021	44	July 14	465	LI, AGB	RGB and multispectral
	57	July 27	614	LI, AGB	RGB and multispectral
	70	August 9	763	LI, AGB	RGB and multispectral
	94	September 2	1045	LI, AGB	RGB and multispectral
	116	September 24	1252	LI, AGB	RGB and multispectral
	144	October 25	1458	Lint Yield	RGB
2022	43	June 7	372	LI, AGB	RGB and multispectral
	59	June 23	569	LI, AGB	RGB and multispectral
	75	July 9	768	LI, AGB	RGB and multispectral
	100	August 3	1060	LI, AGB	RGB and multispectral
	114	August 17	1220	LI, AGB	RGB and multispectral
	150	September 22	1588	Lint Yield	RGB

DAP, Days after planting; GDDs, Growing degree days; LI, Light Interception; AGB, Above-ground biomass; UAV, Unmanned Aerial Vehicle; RGB, Red Green Blue.

#### 2.2.1 Physiological parameters and yield measurements

An AccuPAR LP-80 ceptometer (METER Environment, Pullman, WA) was used for light interception measurements from the middle two rows of each plot between 1000 and 1400 h under cloudless conditions. An integrated linear sensor and an attached external sensor to a tripod stand were used to measure below-canopy photosynthetically active radiation (PAR _below_) and above-canopy photosynthetically active radiation (PAR_above_), respectively. The PAR_below_ is an average value from the linear sensor positioned perpendicular and parallel to the row. These values were used to calculate the fraction of intercepted photosynthetically active radiation (IPAR_f_) on a given day using equation 2.


(2) $${\text{IPAR}}_{\text{f}}=({\text{PAR}}_{\text{above}}–{\text{PAR}}_{\text{below}})/{\text{PAR}}_{\text{above}}$$

Furthermore, cumulative incident PAR from planting until a specific sampling date was calculated by summing incident daily PAR (assuming PAR is 45% of solar radiation) for all days included in the defined time frame. Finally, cumulative IPAR (MJ m^-2^) at the sampling date was the product of IPAR_f_ and cumulative incident PAR.

Above-ground dry biomass in 2021 was obtained by harvesting all plants from a one-meter length of the row from one of the middle two rows of each plot, while in 2022, the sample size was increased to a two-meter length of the row. The fresh samples were dried in a forced-air oven at 80°C for 72 hours and then weighed to obtain above-ground dry biomass in g m^-2^.

Radiation use efficiency (RUE) (g MJ^-1^) was determined by dividing the change in above-ground dry biomass (g m^-2^) by the change in cumulative IPAR (MJ m^-2^), where the first sampling date was taken as a reference point as shown in equation 3.


(3) $$\text{RUE}=(\text{Dry }{\text{biomass}}_{\text{n}}-\text{Dry }{\text{biomass}}_{1})/(\text{Cumulative }{\text{IPAR}}_{\text{n}}-\text{Cumulative }{\text{IPAR}}_{1})$$

where, dry biomass *_1_* and cumulative IPAR *_1_* are the reference measurements of the first sampling date, and *n* represents each subsequent sampling date in the season.

Further, the harvest index (HI) for each plot was calculated as the ratio of lint yield (kg ha^−1^) to the highest above-ground dry biomass obtained during the season (kg ha^-1^). For lint yield (kg ha^-1^), the middle two rows from each plot during both years were mechanically harvested using a two-row spindle cotton picker (John Deere 9930 (John Deere, Moline, IL)), and the harvested seed-cotton was ginned at the University of Georgia MicroGin (Li et al., 2011) to obtain a realistic measure of gin turnout and lint yield (kg ha^-1^).

#### 2.2.2 UAV data collection and image processing

Both RGB and multispectral imagery were acquired during 1000 to 1400 h using a flight altitude of 45 m. The RGB aerial imagery was acquired using an integrated RGB sensor on DJI Phantom^™^ Pro 4 V2.0 (Shenzhen, China) in 2021 and on DJI Mavic^™^ Air 2 (Shenzhen, China) in 2022. The DJI Mavic Air 2 was used on the September 22, 2022 sampling date (prior to harvesting) because of its higher resolution. Multispectral images were acquired during both years using a MicaSense RedEdge-MX^™^ (Seattle, WA) sensor mounted on a DJI Inspire^™^ 2 (Shenzhen, China) UAV platform. Detailed information on the sensors and the flight settings used for aerial imagery data collection is provided in **Table 2**. Prior to any data collection each year, ground control points (GCPs) were placed at four corners of the field and were georeferenced using a handheld Trimble GNSS Unit (Sunnyvale, CA) with a GPS/GNSS RTK correction ( ± 0.50 cm) enabled. All flights were created and implemented using the Pix4Dcapture^®^ software (Pix4D, Lausanne, Switzerland).

**Table 2:** Flight plan details and technical specifications for the RGB and multispectral sensors used for aerial imagery collection in the study sites for the 2021 and 2022 growing seasons.

Flight details and specifications	DJI’s Phantom^™^ Pro 4 V2.0	DJI’s Mavic^™^ Air 2	MicaSense RedEdge-MX^™^
Flight Altitude	45 m	45 m	45 m
Spatial Resolution	1.33 cm	1.62 cm	3.29 cm
Image Overlap	Side 70% Front 80%	Side 70% Front 80%	Side 80% Front 80%
Flight Speed	4.11 m s^-1^	4.91 m s^-1^	3.81 m s^-1^
Sensor	1-inch CMOS; 20 Megapixel	1/2-inch CMOS; 48 Megapixel	RedEdge-MX sensor
Bits per pixel	16	16	12
Spectral Range	RGB	RGB	Blue (475 nm ± 20 nm) Green (560 nm ± 20 nm) Red (668 nm ± 10 nm) Red-edge (717 nm ± 10 nm) Near Infrared (840 nm ± 40 nm)

After each UAV flight, all images were downloaded and processed in the Pix4Dmapper^®^ software (Pix4D, Lausanne, Switzerland) using the GCPs and pre-flight calibration to create one orthorectified mosaic images per bands for each sampling date. The Pix4Dmapper^®^ software uses modified structure-from-motion (SFM) approach to create the orthorectified mosaic images. For radiometric calibration of multispectral images, before each flight the MicaSense RedEdge-MX™ multispectral sensor was used to capture reference images of a MicaSense Calibrated Reflectance Panel provided by the manufacturer. These captured reference images for each multispectral band are used act as an input in Pix4Dmapper processing options to perform radiometric calibration and correction for each of the five bands.

#### 2.2.3 Reflectance values and image feature extraction

Orthorectified mosaic multispectral images were used to extract raw reflectance values for each plot. The reflectance values for each band (red, green, blue, red-edge, and near-infrared) for all sampling dates were extracted in ArcMap^®^ 10.7.1 (ESRI, Redlands, CA). Cotton canopy and soil were segmented in each image using a classification index: multiplication of the Normalized Difference Vegetation Index (NDVI) and Excessive Greenness Index (ExG) (Moghimi et al., 2018). A global threshold (pixel values greater than 0.02) was applied to the classified image to obtain a binary mask layer to separate the canopy from bare soil. The binary mask layer was applied to the orthorectified mosaic image for each reflectance band. A polygon specifying the region of interest (ROI) – the middle two rows of each plot – was created and used to extract the averaged reflectance values for all multispectral bands for each plot. The reflectance values for each band were further used to compute several VIs as shown in **Table 3** using Microsoft Excel^®^ (Redmond, WA).

**Table 3:** List of 20 different vegetation indices (VIs) computed in this study from the raw RGB and Multispectral bands.

Vegetation Indices	Formulation	References
ExG (Excessive Greenness Index)	(2×G)−R−B	Woebbecke et al. (1995)
NDVI (Normalized Difference Vegetation Index)	(NIR−R)/(NIR+R)	Rouse (1974)
GNDVI (Green Normalized Difference Vegetation Index)	(NIR−G)/(NIR+G)	Gitelson et al. (2003a)
NDRE (Normalized Difference Red Edge Index)	(NIR−RE)/(NIR+RE)	Gitelson and Merzlyak (1997)
RVI (Ratio Vegetation Index)	NIR/R	Tucker (1979)
SCCCI (Simplified Canopy Chlorophyll Content Index)	NDRE/NDVI	Raper and Varco (2014)
SAVI (Soil Adjusted Vegetation Index)	(1 + 0.5)((NIR−R)/(NIR+R+0.5))	Huete (1988)
EVI (Enhanced Vegetation Index)	(2.5×NIR−R)/(NIR+6×R−7.5×B+1)	Huete et al. (2002)
EVI2 (Enhamced Vegetation Index2)	(2.5×NIR−R)/(NIR+2.5×R+1)	Jiang et al. (2008)
MSAVI (Modified Soil Adjusted Vegetation Index)	[(2NIR+1)−√((2NIR+1)^2^−8(NIR−R))]/2	Qi et al. (1994)
VARI (Visible Atmospherically Resistant Index)	(G−R)/(G+R−B)	Gitelson et al. (2002)
WDRVI (Wide Dynamic Range Vegetation Index)	(0.2×NIR−R)/(0.2×NIR+R)	Gitelson (2004)
RECI (Rededge Chlorophyll Index)	(NIR/RE)-1	Gitelson et al. (2003b)
RE/R (Redegde to Red ratio)	RE/R	Ballester et al. (2013)
NIR/G (Near infrared to Green ratio)	NIR/G	Sripada et al. (2005)
RGBVI (Red Green Blue Vegetation Index)	(G−B×R)/(G^2^ +(B×R))	Bendig et al. (2015)
GRVI (Green Red Vegetation Index)	(G−R)/(G+R)	Falkowski et al. (2005)
TCARI (Transformed Chlorophyll Absorption Reflectance Index)	3[(RE−R)−0.2(RE−G)×(RE/R)]	Haboudane et al. (2002)
OSAVI (Optimized Soil Adjusted Vegetation Index)	(1 + 0.16)[(NIR−R)/(NIR+R+0.16)]	Rondeaux et al. (1996)
TCARI/OSAVI	TCARI/OSAVI	Haboudane et al. (2002)

R, G, B, RE, and NIR represents Red, Green, Blue, Red-edge, and Near-infrared bands, respectively, and were obtained from multispectral imagery collected at different sampling dates during the 2021 and 2022 season.

RGB images taken immediately before harvest during each year were used to compute cotton fiber index (CFI) values for each plot (Huang et al., 2016; Feng et al., 2020). CFI estimates the total open cotton bolls in an ROI and is calculated using equation 4.


(4) CFI=Number of white pixels in the ROI/Total number of pixels in the ROI

**Figure 2:**
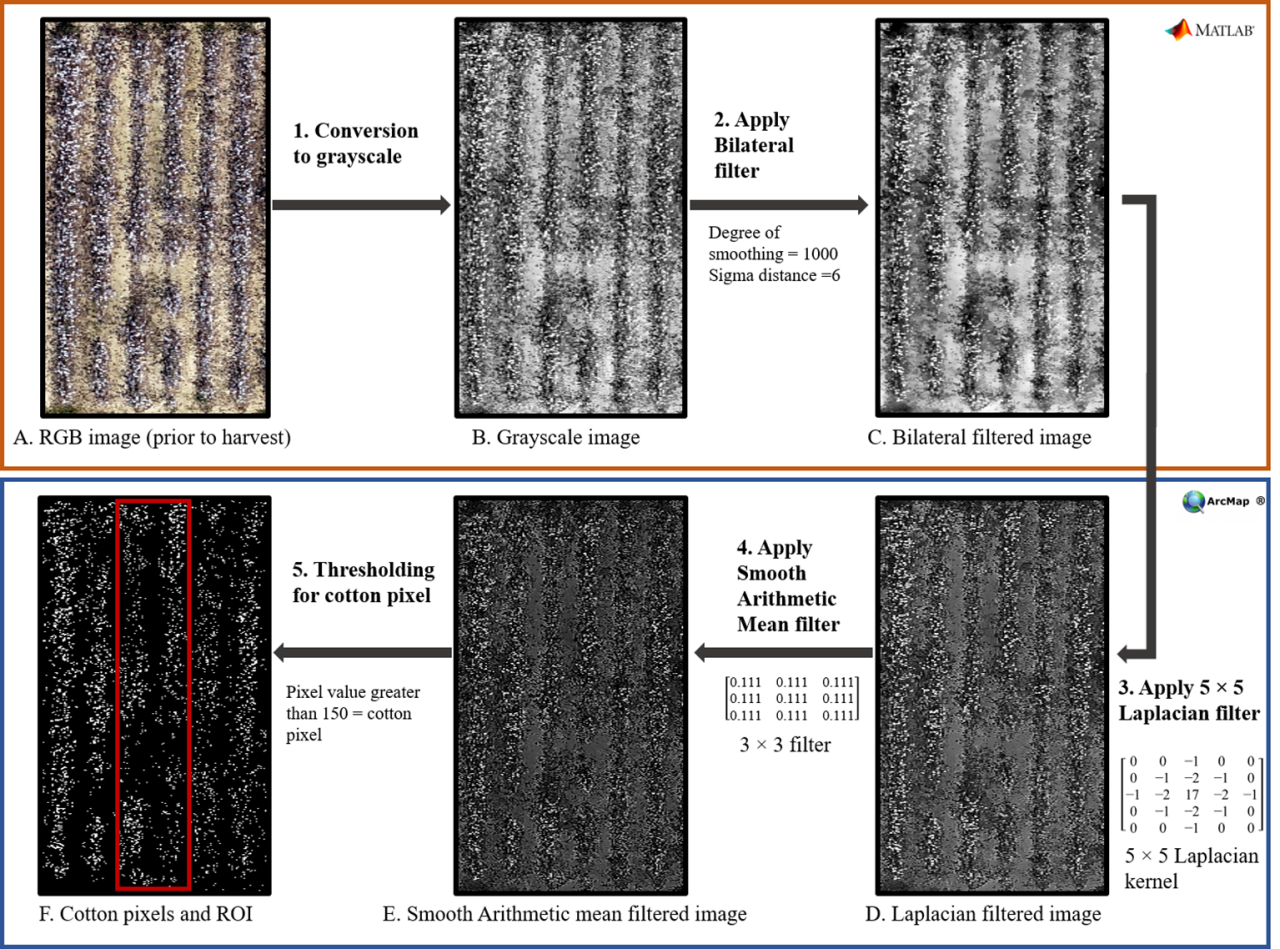
Flowchart to illustrate the different steps (1-5) followed for computing cotton fiber index (CFI) from aerial RGB images using various image filtering and enhancing tools in Matlab^®^ and ArcMap^®^. The top orange box (Steps 1 and 2; Images **A–C**) shows the steps performed in Matlab^®^, and the bottom blue box (Steps 3, 4, and 5; Images D–F) shows the steps performed in ArcMap^®^.

As Feng et al. (2020) and Huang et al. (2016) suggested, a simple global threshold to RGB image was unable to completely differentiate white cotton pixels from background pixels of defoliated vegetation and soil. Therefore, a series of image filtering and image enhancing techniques (**Figure 2**) were performed in MATLAB^®^ 2022b 9.13.0 (The MathWorks Inc., Natick, MA) and ArcMap^®^ 10.7.1 (ESRI, Redlands, CA) for accurate detection of white pixels. First, a bilateral filter (**Figure 2**: Step 2), using a non-linear filtering method was applied for smoothing the images to remove background leaves and soil surface noises, while preserving the shapes and high-intensity values of cotton pixels. Then, a 5 x 5 Laplacian filter (**Figure 2**: Step 3) was applied to increase the contrast and sharpness to enhance the edges of white cotton pixels. An additional smoothing filter, with arithmetic mean (**Figure 2**: Step 4), was further utilized to remove the extra noise introduced by the Laplacian filter. Finally, a threshold (pixel values greater than 150) was applied to separate white cotton pixels, and a zonal histogram tool was used to obtain white pixel counts from each ROI to calculate CFI.

#### 2.2.4 Vegetation indices

Based on the previous literature, 20 different vegetation indices (VIs) that showed strong relationships with leaf area index, biomass, chlorophyll content, plant height, canopy cover, and lint yield of cotton were selected and computed using various combinations of different multispectral bands. These indices are presented in **Table 3**. Further, the relationship of these VIs along with the individual multispectral reflectance bands (green, blue, red, red-edge, and near-infrared) with functional yield-governing parameters in cotton were examined.

### 2.3 Model development and statistical analysis

#### 2.3.1 Variable selection

**Figure 3:**
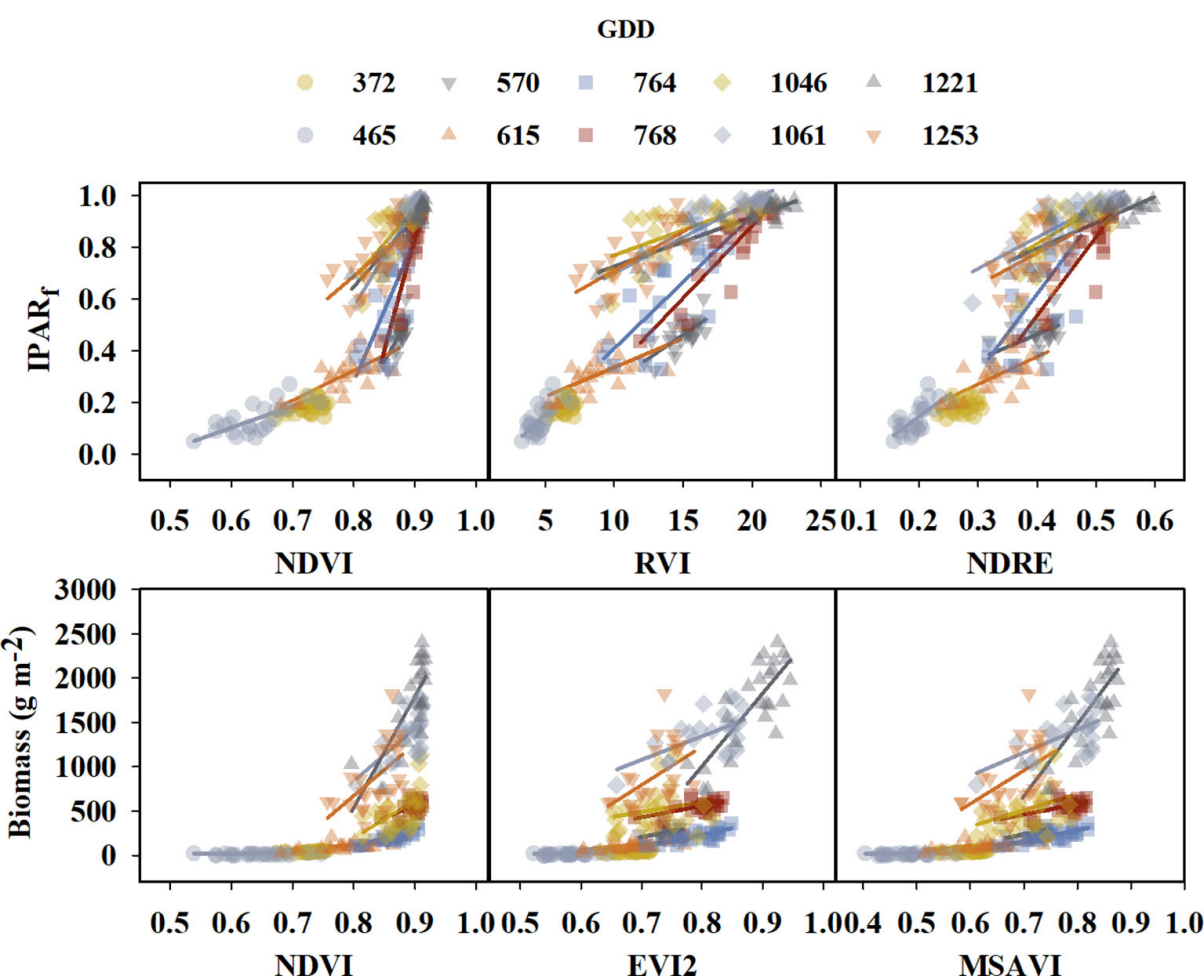
Graphs illustrating linear relationship between the fraction of light interception (IPAR_f_) and above-ground biomass for few selected VIs at different growing degree day (GDD). Different colors and symbols represent different GDDs and are specified in the legend at the top of the graph. Solid lines represent linear regression functions.


As noted in section 2.2.1, the response (independent) variables included measured physiological parameters such as IPAR_f_, above-ground biomass, and HI, as well as RUE*_n_* which is expressed as biomass produced (g) per PAR intercepted (MJ) by the canopy in reference to the first sampling date during the growing seasons, and vegetation index as the predictor (dependent variable). Initially, individual scatter plots were created for 20 different vegetation indices using pooled data from both growing seasons, with IPAR_f_ and above-ground biomass on the Y-axis versus VIs derived from multispectral imagery throughout the growing season on the X axis (**Supplementary Figures 2, 3**). Similarly, for RUE, RUE*_n_* was plotted on the Y-axis versus the average of VIs for the period during which RUE*_n_* was determined on the X-axis (**Supplementary Figure 4**). These plots between IPAR_f_, above-ground biomass, and VIs revealed a non-linear relationship with higher variation for IPAR_f_ and above-ground biomass as VI values increased. The non-linear association between IPAR_f_ and biomass indicated the necessity for an additional predictor in addition to the VIs. In a study to predict above-ground wheat biomass throughout the season, Li et al. (2022) suggested a strong linear relationship of VI’s with biomass at each GDD, and the overall relationship throughout the season evolved with GDD. Similarly, changes were seen in our study where the slope and intercept of standard linear relationships between IPAR_f_ or above-ground biomass and VIs changed at different GDD throughout the season (**Figure 3**). Therefore, along with VI, GDD was added as a dependent variable (predictor) of IPAR_f_, and biomass throughout the season in the model as shown in equation (4). In contrast, the association between RUE*_n_* and the average of VIs was found to be linear with constant variance and the average of VI during a specified time in the season was used as a single predictor of RUE (equation 5).

(4) $${\text{IPAR}}_{\text{f}}\text{ or above-ground biomass}=f(\text{instantaneous VI and GDD})$$

(5) $${\text{RUE}}_{n}=f(\text{average of VI within the RUE calculation period})$$

#### 2.3.2 Model selection

For model development, generalized linear regression (GLR) modeling was performed which assumes that the response variables can have a variety of distributions depending on their characteristics and the predictors. Generalized linear models (GLMs) from GLR consist of three components: the random component, the systematic component, and the link function to connect the random and systematic components (Nelder and Wedderburn, 1972; Dunn and Smyth, 2018). The random component represents the distribution of the response variable given the predictors. IPAR_f_ is a fractional value that always lies between 0 and 1. As a result, the beta distribution with a logistic link function, which is effective for continuous data ranging from 0 to 1, was chosen as a random component for IPAR_f_. Biomass throughout the season is a positive value; therefore, gamma distribution that includes an exponential relationship with a log link function was chosen as a random component for above-ground biomass. RUE seems to be linearly related to the average of VIs with constant variance; therefore, normal distribution with identity link function (equivalent to standard linear regression) was chosen as the random component. The systematic component of GLM represents the linear predictors which were identified in equations 4 and 5.

#### 2.3.3 Model validation

The 2021 and 2022 growing seasons had different planting and harvesting times, resulting the cotton plants with seasonal variances in growth and development that can be captured in the UAV imagery. As a result of distinct growth patterns, all data collected throughout the season from both growing seasons for IPAR_f_, RUE, and biomass were pooled together, and the pooled data were randomly stratified by the sampling date in JMP^®^ Pro 16.0.0 (SAS, Cary, NC) where 60% of the data (training data) was used for model generation and the remaining 40% (validation data) for cross-validation. Outliers were identified using histogram, boxplot, and interquartile range methods and were removed from the data before generating training models. There were only two data points identified as outliers, which could have been introduced by noise during reflectance measurement. Cross-validation was performed for each model by plotting predicted versus measured fit for the validation dataset. The models generated from GLR using different VIs were ranked based on the generalized coefficient of determination (R^2^), Akaike information criterion value (AICc), and Bayesian information criterion value (BIC) for training dataset, and the coefficient of determination (R^2^cv) and root mean sum square error (RMSEcv) for cross-validation. Higher R^2^ and R^2^cv values along with lower AICc, BIC, and RMSEcv values were viewed as better model performance qualities.

HI was estimated as the ratio of predicted lint yield to predicted above-ground biomass. For lint yield estimation, standard linear regression was performed between the machine-harvested lint yield and CFI. The cross-validation for lint yield modeling could not be performed due to the limited number of data points (N=50). Finally, the estimated HI was compared to the observed HI (Section 2.3.1) to obtain the coefficient of determination (R^2^) and RMSE values. All modeling and statistical analyses were performed in JMP^®^ Pro 16.0.0 (SAS, Cary, NC) and graphs were prepared using Sigma Plot 14.0 (Systat Software Inc., San Jose, CA).

## 3 Results

### 3.1 Fraction of intercepted photosynthetically active radiation

**Figure 4:**
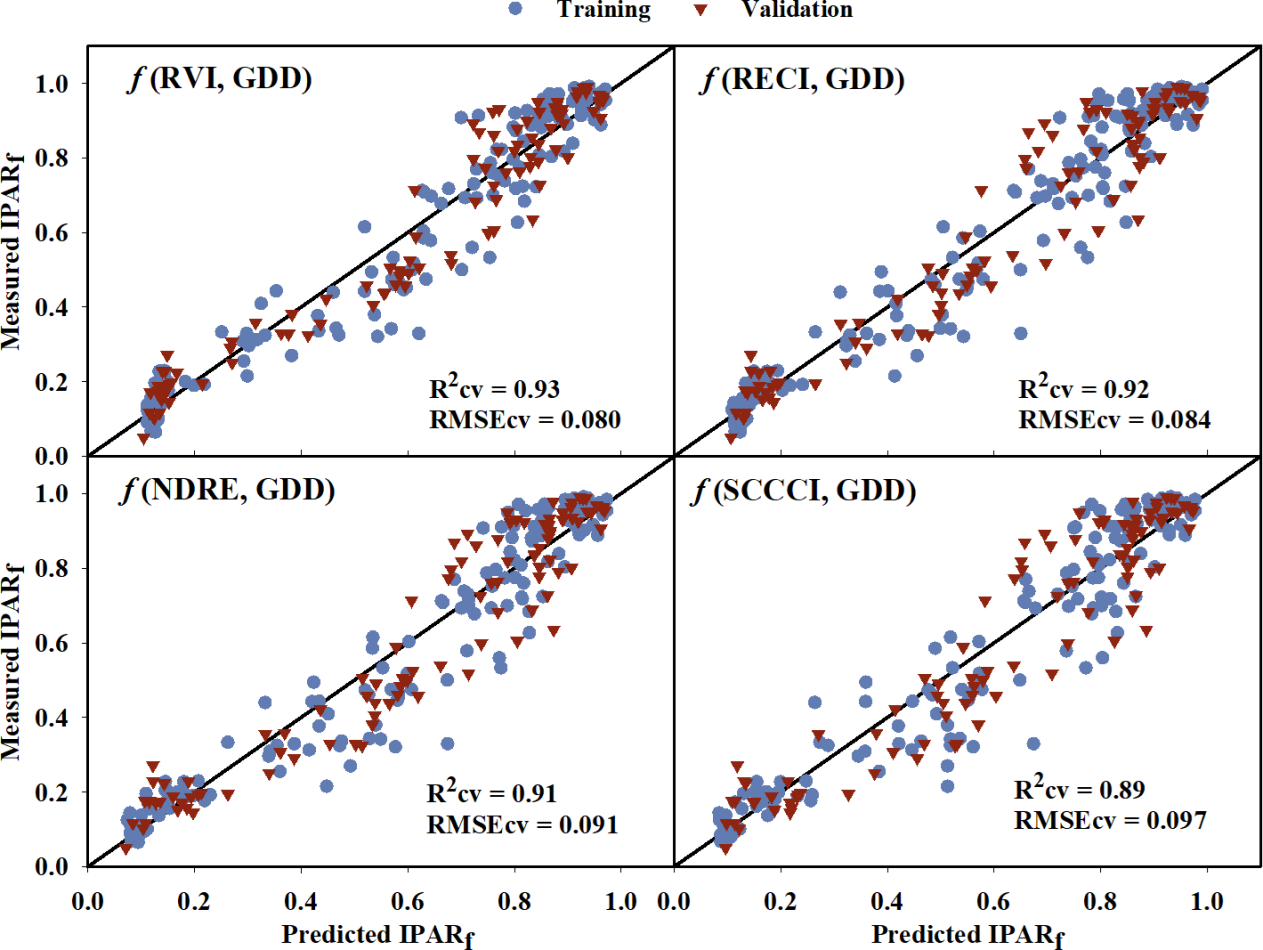
Predicted versus measured fraction of Intercepted Photosynthetically Active Radiation (IPAR_f_) for the four models that performed the best in predicting IPAR_f_. Blue circles represent the training data, and red triangles represent the validation data. R^2^cv and RMSEcv represent the coefficient of determination and root mean sum square error, respectively, for cross-validation. The diagonal line is a reference line with a slope equal to 1. GDD, Growing Degree Days; RVI, Ratio Vegetation Index; RECI, Red-edge Chlorophyll Index; NDRE, Normalized Difference Red-edge Index; SCCCI, Simplified Canopy Chlorophyll Content Index.

The IPAR_f_ models generated using training data had generalized R^2^ values ranging from 0.66 to 0.90 (**Table 4**). The model equations are provided in the **Supplementary Table 1**. Upon comparing the model performance for both training and cross-validation, RVI, RECI, NDRE, and SCCCI were the top four VIs with the highest R^2^ and R^2^cv values, and lowest AICc, BIC, and RMSEcv values. The predicted IPAR_f_ from these four models was able to explain 89% to 93% of the variation in measured IPAR_f_ with RMSEcv ranging from 0.080 to 0.097 during cross-validation (**Figure 4**). Compared to the VIs, models from near-infrared and red reflectance bands explained 85% and 87% of measured IPAR_f_; however, the RMSEcv was higher for these raw bands (0.139, and 0.115, respectively).

**Table 4:** Model performance parameters for predicting fraction of Intercepted Photosynthetically Active Radiation (IPAR_f_) using different vegetation indices (VIs) and raw reflectance bands for the studies conducted in the 2021 and 2022 growing seasons (model equations provided in **Supplementary Table 1**).

VIs	R^2^	AICc	BIC	R^2^cv	RMSEcv
RVI	0.90	-359.43	-347.69	0.93	0.080
RECI	0.89	-351.13	-339.39	0.92	0.084
NDRE	0.88	-332.31	-320.57	0.91	0.091
SCCCI	0.87	-318.60	-306.87	0.89	0.097
WDRVI	0.85	-297.40	-285.66	0.90	0.099
MSAVI	0.83	-278.31	-266.58	0.91	0.100
NIR/G	0.86	-307.54	-295.80	0.88	0.101
OSAVI	0.82	-275.32	-262.59	0.90	0.103
SAVI	0.81	-261.05	-249.31	0.90	0.105
VARI	0.82	-269.98	-258.25	0.90	0.106
NDVI	0.82	-272.21	-260.47	0.88	0.107
RE/R	0.82	-270.38	-258.65	0.89	0.107
EVI2	0.79	-246.48	-234.74	0.89	0.109
EVI	0.79	-247.96	-236.22	0.89	0.110
GRVI	0.80	-257.86	-246.12	0.89	0.111
REDEDGE	0.83	-271.05	-259.37	0.84	0.111
GNDVI	0.81	-265.86	-254.13	0.86	0.113
RED	0.81	-255.02	-243.34	0.87	0.115
RGBVI	0.79	-247.66	-235.93	0.87	0.115
TCARI	0.77	-245.89	-234.15	0.87	0.116
TCARI/OSAVI	0.78	-240.74	-229.01	0.86	0.119
GREEN	0.77	-227.54	-215.86	0.82	0.126
BLUE	0.73	-207.82	-196.14	0.82	0.134
NIR	0.70	-191.24	-179.56	0.85	0.139
ExG	0.66	-178.21	-166.48	0.77	0.148

R^2^, AICc, and BIC represent the generalized coefficient of determination, Akaike Information Criterion and Bayesian Information Criterion values, respectively, for the training data (N = 149). R^2^cv and RMSEcv represent the coefficient of determination and root mean sum square error, respectively, for cross-validation (N = 100).

### 3.2 Above-ground biomass

**Figure 5:**
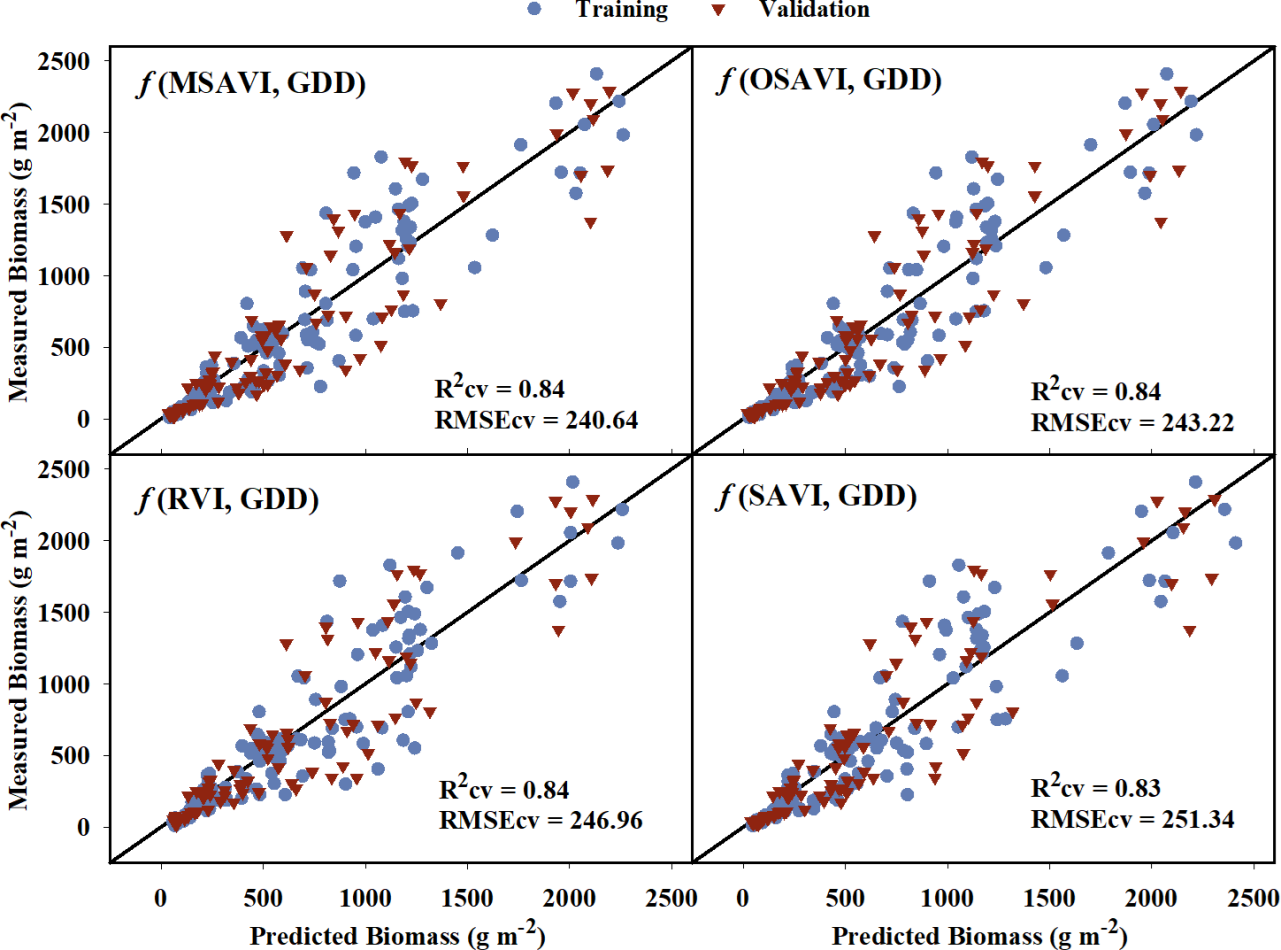
Predicted versus measured above-ground biomass for the four models that performed the best in predicting above-grond biomass models. Blue circles represent the training data, and red triangles represent the validation data. R^2^cv and RMSEcv represent the coefficient of determination and root mean sum square error, respectively, for cross-validation. The diagonal line is a reference line with a slope equal to 1. GDD, Growing Degree Days; MSAVI, Modified Soil Adjusted Vegetation Index; OSAVI, Optimized Soil Adjusted Vegetation Index; RVI, Ratio Vegetation Index; SAVI, Soil Adjusted Vegetation Index.

For above-ground biomass, models developed from the training dataset had generalized R^2^ values ranging from 0.69 to 0.87 indicating a moderate to strong relationship with the VIs (**Table 5**). These model equations are provided in **Supplementary Table 2**. Based on the model performance, MSAVI, OSAVI, RVI, and SAVI were the four VI-based models with the highest R^2^ and R^2^cv values, and the lowest AICc, BIC, and RMSEcv values. The estimated above-ground biomass from these models was able to explain 83-84% of variation with RMSEcv ranging from 240.64 to 251.34 g m^-2^ during cross-validation (**Figure 5**). GNDVI and NIR/G had the highest R^2^ values of 0.86 and 0.87, respectively, during training, but the R^2^ values reduced to 0.75 and 0.76, respectively during cross-validation. Out of the five raw bands, the model with the NIR band was able to explain 79% of the variation in the measured above-ground biomass during cross-validation.

**Table 5:** Model performance parameters for predicting above-ground biomass using different vegetation indices (VIs) and raw reflectance bands for the studies conducted in the 2021 and 2022 growing seasons (model equations provided in **Supplementary Table 2**).

Vis	R^2^	AICc	BIC	R^2^cv	RMSEcv
MSAVI	0.87	1896.01	1907.75	0.84	240.64
OSAVI	0.87	1895.28	1907.01	0.84	243.22
RVI	0.86	1904.01	1915.75	0.84	246.96
SAVI	0.86	1909.57	1921.31	0.83	251.34
EVI	0.84	1924.82	1936.56	0.82	260.19
EVI2	0.84	1924.57	1936.30	0.82	260.26
WDRVI	0.86	1906.92	1918.66	0.81	263.85
NIR	0.82	1910.56	1922.24	0.79	267.15
NDVI	0.85	1917.75	1929.48	0.79	278.17
NDRE	0.85	1915.79	1927.53	0.79	280.39
VARI	0.80	1958.09	1969.83	0.78	284.61
RECI	0.84	1925.15	1936.89	0.78	286.98
GRVI	0.80	1962.05	1973.78	0.77	290.94
SCCCI	0.84	1928.49	1940.23	0.77	293.92
NIR/G	0.87	1896.81	1908.55	0.76	295.95
RE/R	0.82	1939.01	1950.75	0.76	300.85
RED	0.82	1903.43	1915.11	0.70	303.97
GNDVI	0.86	1910.01	1921.75	0.75	305.15
TCARI	0.82	1942.01	1953.75	0.70	330.58
TCARI/OSAVI	0.82	1944.09	1955.83	0.70	333.46
RGBVI	0.78	1971.23	1982.97	0.69	339.64
BLUE	0.78	1935.68	1947.36	0.62	344.66
REDEDGE	0.77	1943.90	1955.58	0.61	348.08
GREEN	0.78	1938.76	1950.44	0.56	362.22
ExG	0.69	2022.66	2034.39	0.56	405.25

R^2^, AICc, and BIC represent the generalized coefficient of determination, Akaike Information Criterion and Bayesian Information Criterion values, respectively, for the training data (N = 147). R^2^cv and RMSEcv represent the coefficient of determination and root mean sum square error, respectively, for cross-validation (N = 100).

### 3.3 Radiation use efficiency

**Figure 6:**
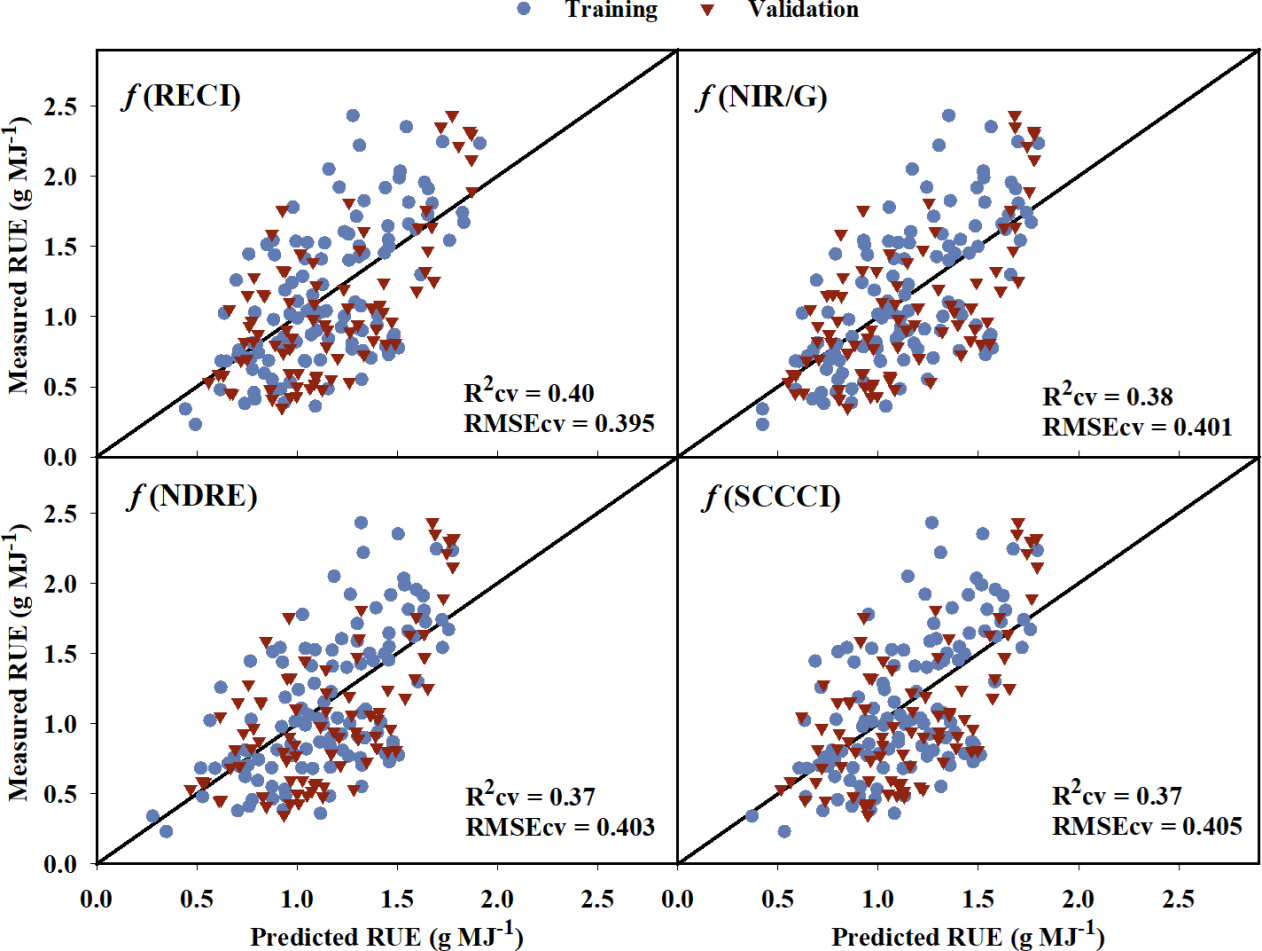
Predicted versus measured radiation use efficiency (RUE) for the the four models that performed the best in predicting RUE. Blue circles represent the training data, and red triangles represent the validation data. R^2^cv and RMSEcv represent the coefficient of determination and root mean sum square error, respectively, for cross-validation. The diagonal line is a reference line with a slope equal to 1. RECI, Red-edge Chlorophyll Index; NIR/G, Near infrared to Green Ratio; NDRE, Normalized Difference Red-edge Index; SCCCI, Simplified Canopy Chlorophyll Content Index.

The models created using training data to predict RUE explained 2% to 43% of the variation in RUE indicating a poor to low relationship of VIs to the RUE (**Table 6**). The model equations are provided in **Supplementary Table 3**. While some of the VIs such as WDRVI, OSAVI, MSAVI, and TCARI explained as much as 43% variation in the RUE for the training data, they did not perform well during validation (Rcv = 25-33%). RECI, NIR/G, NDRE, and SCCCI performed best during training and cross-validation with lower AICc, BIC, and RMSEcv values, and the highest R^2^cv value. A plot of predicted versus measured RUE using validation data (**Figure 6**) showed that the above-mentioned models explained 37% to 40% of the variation in cotton RUE with around 0.4 g MJ^-1^ RMSEcv. Among the five raw reflectance bands, the RUE prediction model using the red-edge and green bands explained 37% and 34%, respectively, of variation in RUE during cross-validation.

**Table 6:** Model performance parameters for predicting Radiation Use Efficiency (RUE) using different vegetation indices (VIs) and raw reflectance bands for the studies conducted in the 2021 and 2022 growing seasons (model equations provided in **Supplementary Table 3**).

VIs	R^2^	AICc	BIC	R^2^cv	RMSEcv
RECI	0.38	123.82	131.84	0.40	0.395
NIR/G	0.41	117.46	125.48	0.38	0.401
NDRE	0.39	121.16	129.17	0.37	0.403
SCCCI	0.35	128.72	136.74	0.37	0.405
REDEDGE	0.30	137.61	145.63	0.37	0.402
GNDVI	0.43	113.26	121.28	0.36	0.406
RVI	0.42	115.29	123.31	0.35	0.409
GREEN	0.36	127.07	135.08	0.34	0.412
WDRVI	0.43	114.37	122.39	0.33	0.417
OSAVI	0.43	114.40	122.42	0.32	0.420
EVI	0.42	115.74	123.76	0.32	0.420
NDVI	0.41	117.03	125.04	0.32	0.420
RED	0.40	120.18	128.2	0.32	0.420
SAVI	0.43	114.00	122.02	0.31	0.421
MSAVI	0.43	113.64	121.66	0.31	0.423
EVI2	0.42	114.91	122.93	0.31	0.424
NIR	0.37	125.27	133.29	0.26	0.438
TCARI/OSAVI	0.42	115.41	123.16	0.25	0.439
VARI	0.37	125.89	133.91	0.25	0.441
TCARI	0.43	113.24	121.26	0.24	0.444
RE/R	0.41	118.24	126.26	0.23	0.447
GRVI	0.35	129.39	137.41	0.22	0.452
BLUE	0.30	137.59	145.61	0.19	0.458
RGBVI	0.34	131.01	139.03	0.18	0.460
ExG	0.02	175.65	183.67	0.06	0.493

R^2^, AICc, and BIC represent the coefficient of determination, Akaike Information Criterion and Bayesian Information Criterion values, respectively, for the training data (N = 115). R^2^cv and RMSEcv represent the coefficient of determination and root mean sum square error, respectively, for cross-validation (N = 82).

**Figure 7:**
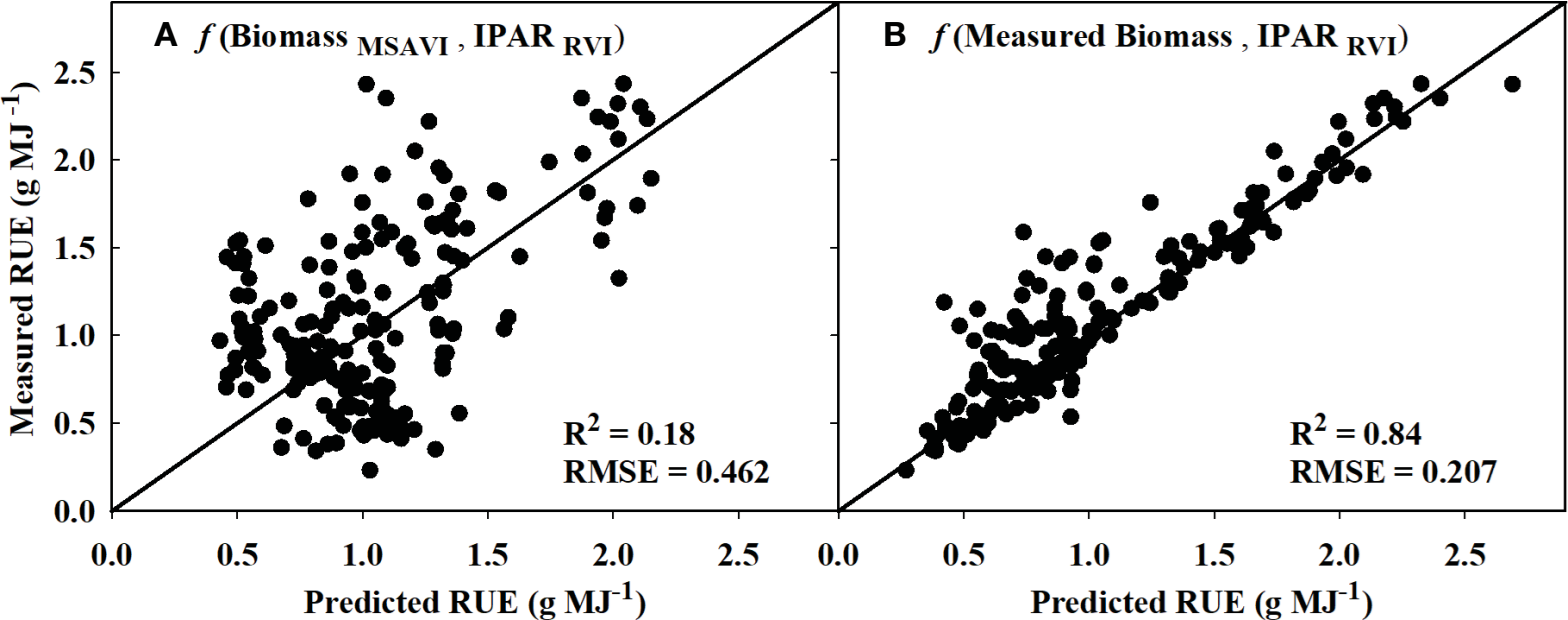
Predicted versus measured plot for the radiation use efficiency (RUE) values obtained using **(A)** MSAVI-based biomass model/RVI-based IPAR model and **(B)** measured biomass/RVI-based IPAR model. R^2^ and RMSE represent the coefficient of determination and root mean sum square error. The diagonal line is a reference line with a slope equal to 1. IPAR, Intercepted Photosynthetically Active Radiation; MSAVI, Modified Soil Adjusted Vegetation Index; RVI, Ratio Vegetation Index.

Based on the model identified for IPAR_f_ and biomass in sections 3.1 and 3.2, the models that performed the best during validation were used to derive RUE using equation 3. The estimated RUE from MSAVI-based biomass model and RVI-based IPAR_f_ model only explained 18% of the variation in measured RUE with an RMSE of 0.462 (**Figure 7A**). However, replacing the MSAVI-based biomass model with the ground-measured biomass increased the prediction accuracy to 84% with the RMSE of 0.207 (**Figure 7B**).

### 3.4 Lint yield and harvest index

**Figure 8:**
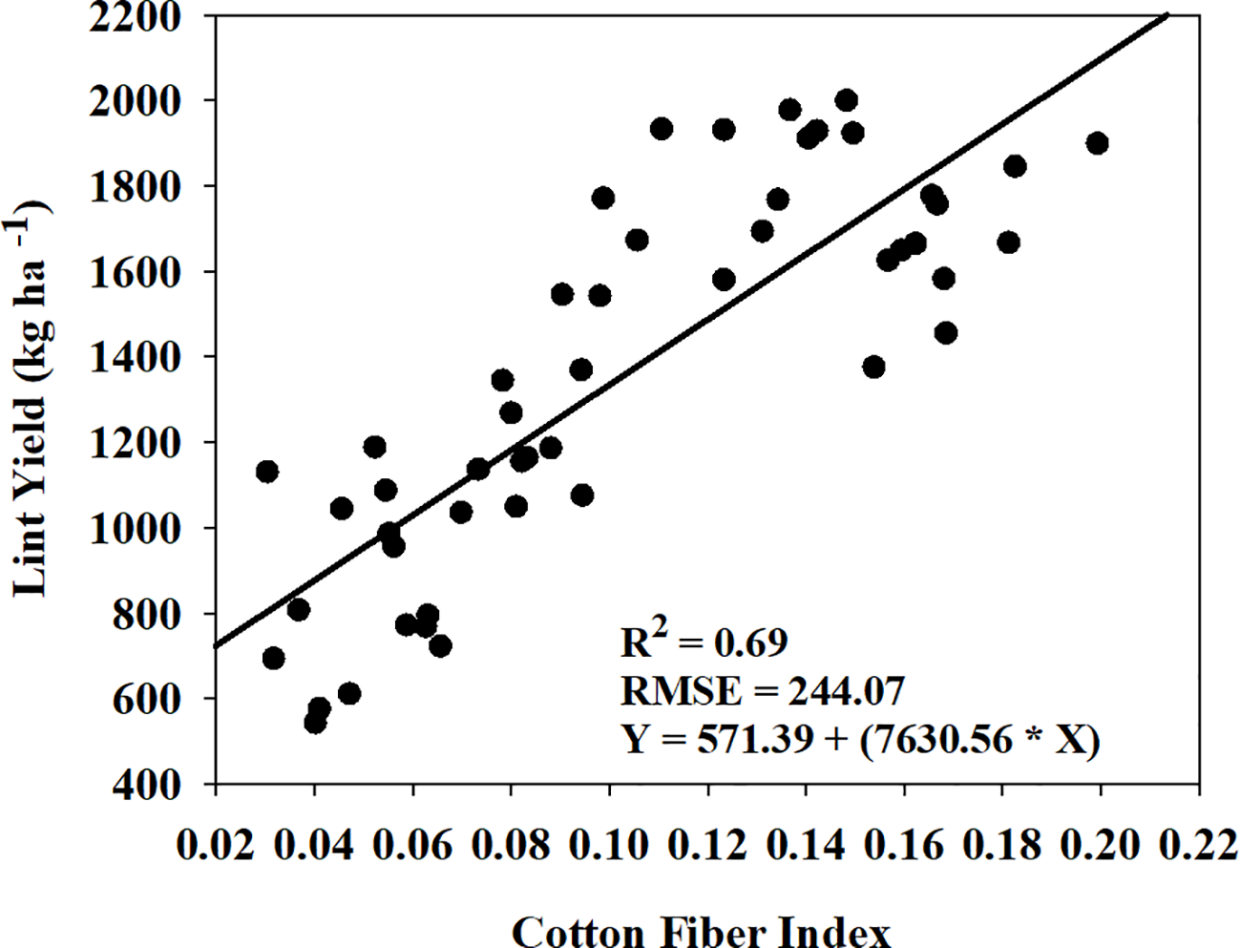
Relationship and prediction equation between machine-harvested lint yield and cotton fiber index (CFI) for both the 2021 and 2022 growing seasons. R^2^ and RMSE represent the coefficient of determination and root mean square error, respectively, for the relationship. In the given equation, Y represents lint yield and X represents CFI.

**Figure 9:**
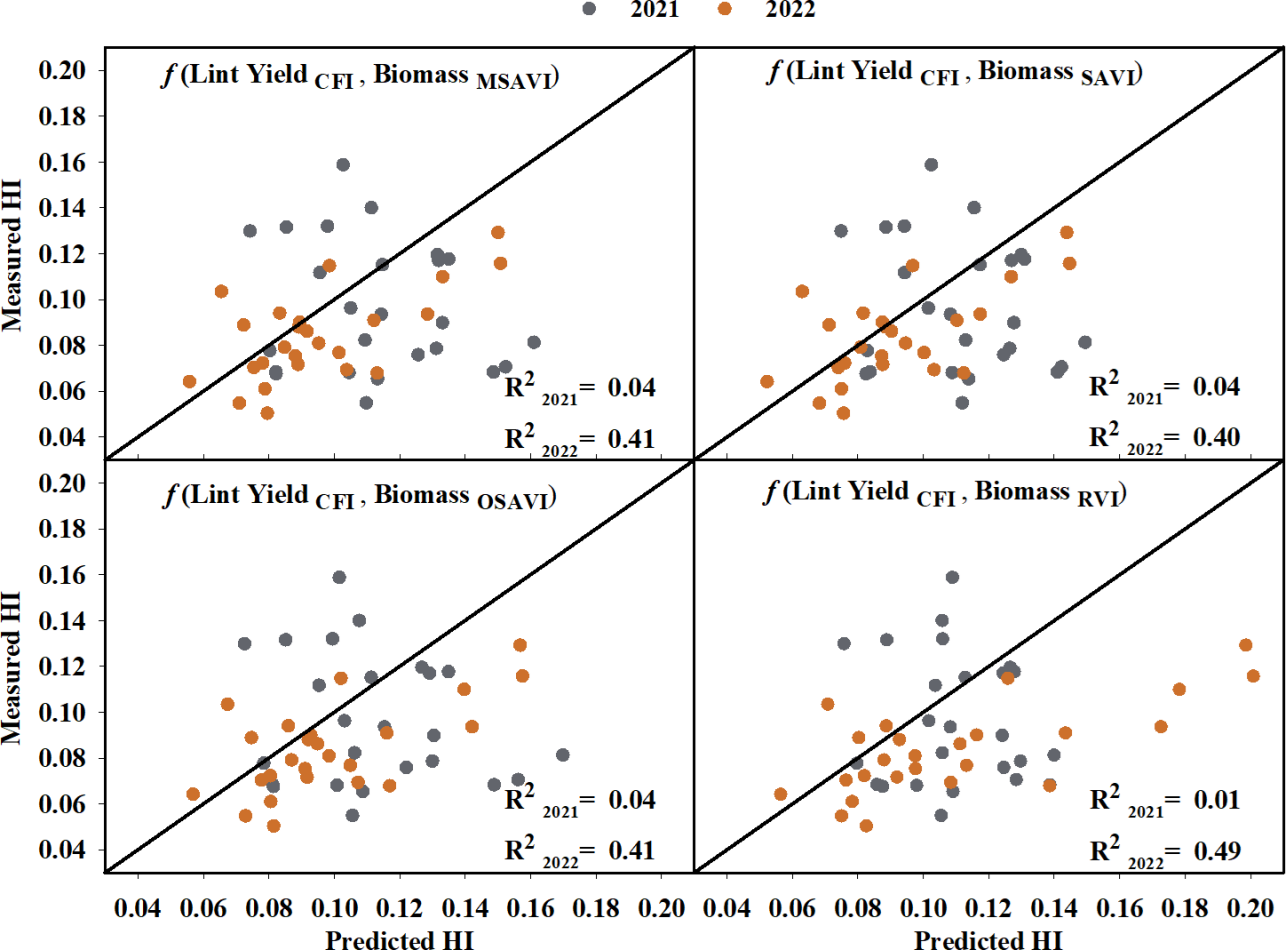
Predicted versus measured harvest index (HI) using CFI-based lint yield and the highest seasonal biomass obtained from the four models that performed the best in predicting above-ground biomass. Grey circles represent the 2021 growing season, and orange circles represent the 2022 growing season. R^2^_2021_ and R^2^_2022_ represent the coefficient of determination for predicted versus measured regression for the 2021 and 2022 growing season, respectively. The diagonal line is a reference line with a slope equal to 1. CFI, Cotton Fiber Index; MSAVI, Modified Soil Adjusted Vegetation Index; OSAVI, Optimized Soil Adjusted Vegetation Index; RVI, Ratio Vegetation Index; SAVI, Soil Adjusted Vegetation Index.

Cotton fiber Index (CFI) from UAV-based RGB imagery showed a linear relationship with machine-harvested lint yield and explained 69% of the variation (RMSE = 244.07 kg ha^-1^) in the lint yield (**Figure 8**). However, the estimated harvest index (HI) utilizing the CFI-based lint yield model and the four models that performed the best in predicting above-ground biomass (**Figure 9**) wasn’t significant (p-value > 0.05) in explaining measured HI. A plot of predicted versus measured HI for the two growing seasons (2021 and 2022) (**Figure 9**) shows that the estimated HI was able to explain 40% to 49% of the variation in cotton HI, for the 2022 growing season, while only 1% to 4% for the 2021 growing season.

## 4 Discussion

The yield-driving physiological parameters – intercepted photosynthetically active radiation (IPAR), radiation use efficiency (RUE), and harvest index (HI), are the key contributors to yield in cotton under different management conditions (Conaty and Constable, 2020). However, the current methods of ground measurement for these parameters are resource intensive and require destructive sampling of plants. Remote sensing using UAVs and low-cost sensors have proven to be an efficient approach in estimating crop biophysical traits such as biomass, chlorophyll content, and leaf area index (Gutierrez et al., 2012; Raper and Varco, 2014; Wang et al., 2021). Therefore, this study investigates the use of low-cost and high-resolution UAV-based RGB and multispectral imagery to predict the aforementioned yield-driving physiological parameters [IPAR, RUE, and HI] of cotton.

### 4.1 Estimation of IPAR_f_, RUE, and biomass

The first objective of this study was to develop and validate models to estimate the fraction of IPAR (IPAR_f_), RUE, and biomass of cotton throughout the season using reflectance data (which includes multispectral vegetation indices (VIs) and raw reflectance bands) and growing degree days (GDD). Past research has demonstrated that the relationship between crop biophysical characteristics and VIs can be altered by growth stage (Gutierrez et al., 2012; Li Z. et al., 2022). For example, during cotton growth and development, biomass distribution, specific leaf nitrogen and chlorophyll contents in the canopy change, resulting in varied spectral responses throughout the season (Snider et al., 2021b). Gutierrez et al. (2012) showed the need of separate models to explain above-ground biomass and LAI of cotton plants at different days after planting. In this study, the inclusion of the GDD term as a predictor helped in addressing the above limitation and allowed us to directly account for the changes in the spectral reflectance of cotton plants at different growth stages during the growing season. The proposed model of VIs along with GDD can be used to estimate the IPAR_f_ and aboveground biomass throughout the season.

During cross-validation, the developed models explained up to 93% of the variation in measured IPAR_f_ of the cotton canopy (**Table 4**), where RVI, RECI, NDRE, and SCCCI had the highest coefficient of determination (**Figure 4**). Past studies have relied on either a physical radiative transfer model (Clevers et al., 1989) or hyperspectral VIs (Tan et al., 2013; Tan et al., 2018) to estimate the fraction of light absorption by crop canopies. However, there is limited information on the relationship between multispectral VIs and IPAR_f_. The amount of light intercepted by a cotton canopy is highly influenced by canopy structure and architectural traits such as leaf area index (Brodrick et al., 2013; Bhattacharya, 2019). Multiple studies have reported the association between cotton leaf area index (LAI) and multispectral VIs (Hatfield et al., 1984; Zhao et al., 2007b; Gutierrez et al., 2012), and insights on the relationship between IPAR_f_ and VIs can be drawn from these studies. RVI, the ratio of near-infrared to red reflectance, has been shown to be effective (R^2 ^= 0.69 to 0.93) in explaining cotton LAI (Zhao et al., 2007a; Gutierrez et al., 2012; Chen, 2019). In the visible and near-infrared wavelength region, the near-infrared is reflected by the canopy and is sensitive to green photosynthetically active vegetation, whereas the red band is strongly absorbed by chlorophyll, making RVI a good predictor of LAI (Tucker, 1979). In this study, models based on raw bands of near-infrared and red reflectance were also able to moderately estimate IPAR_f_ throughout the growing season, further highlighting the importance of these two reflectance bands in explaining the variability in IPAR_f_. NDVI, which is also a function of near-infrared and red reflectance bands, has been used to predict cotton LAI and canopy cover (Ritchie et al., 2010; Adams et al., 2021). Guillen-Climent et al. (2012) also reported that out of several VIs, NDVI was best correlated with IPAR_f_ for crops with homogenous canopies such as wheat, maize, and soybean. However, in the current study, NDVI saturated after the third sampling date (800 GDD) with strong absorption of red wavelength light by the canopy (red reflectance moved closer to zero) and couldn’t explain the variation in IPAR_f_ during the latter part of the season. A similar trend of NDVI saturating at high LAI was reported by Chen (2019) and Gutierrez et al. (2012). The other best performing VIs, RECI, NDRE, and SCCCI, are also reported to explain LAI and canopy chlorophyll content. RECI has been found to be closely related to maize LAI (R^2 ^= 0.95), even at higher LAI values (0 to > 6), without reaching saturation. Similarly, RECI performed the best without saturation even at higher GDD in this study. This is because RECI is based on near-infrared and red-edge reflectance, and the red-edge wavelength of light is not strongly absorbed by chlorophyll compared to the red wavelength (Gitelson et al., 2003b). In a similar manner, NDRE, and SCCCI are also based on near-infrared and red-edge reflectance and have been found to be associated with leaf and canopy chlorophyll content (Gitelson et al., 2003a; Raper and Varco, 2014; Ballester et al., 2017), which explains their effectiveness in explaining IPAR_f_ in this study.

The developed models using reflectance data and GDD were able to explain 55% to 84% of the variation in above-ground biomass for the validation dataset (**Table 5**). MSAVI, OSAVI, RVI, and SAVI were the best performing VIs with the highest coefficient of determination (**Figure 5**). The near-infrared band explained the most variation (79%) in above-ground biomass out of the five raw bands. For crops and other vegetation, the red band is highly absorbed by chlorophyll, whereas the near-infrared is reflected from the outer leaf surfaces and spongy mesophyll cells. (Jackson, 1986). The VIs computed based on these two bands, such as NDVI and RVI, have been linked to above-ground biomass previously (Gitelson et al., 2003a; Zhao et al., 2005; Zhao et al., 2007b; Chao et al., 2019). RVI is one of the best VIs to explain above-ground biomass in this study, which is similar to previous findings. NDVI is also widely used VI that has been extensively related to above-ground biomass. However, it tends to saturate early with increasing biomass, and is influenced by soil reflectance, particularly under low vegetation cover (Huete, 1988; Qi et al., 1994; Gutierrez et al., 2012). VIs such as SAVI, MSAVI, and OSAVI are all modifications of NDVI which are more sensitive to changes in vegetation cover and are less influenced by soil reflectance during the lag phase of vegetative growth (Rouse, 1974; Huete, 1988; Qi et al., 1994; Rondeaux et al., 1996), which explain their strong relationship with biomass throughout the season in the current study. Previous studies have also reported RVI (Gutierrez et al., 2012), MSAVI (Brandão et al., 2015), and OSAVI (Zhao et al., 2007b) to be a good predictor of cotton above-ground biomass at early and peak growth stages. However, recent studies have shifted their attention towards use of hyperspectral (Junhua et al., no date) and LIDAR (Sun et al., 2018; Furbank et al., 2019) sensors but these are currently expensive and require extensive data analysis for crop aboveground biomass estimation. In our study, we noticed that with increasing GDD, the estimated above-ground biomass values moved further away from measured values. This source of error and uncertainty in the biomass model could be attributed to the indeterminate growth habit of cotton because the above-ground biomass during the later season is a combination of reproductive and vegetative biomass, and the reflectance data cannot fully capture the reproductive structures in the lower canopy.

The VIs developed from multispectral imagery only explained up to 40% of the variation in cotton RUE (**Table 6**). This could be due to the fact that RUE is affected by various environmental factors and the interaction of different physiological processes, making it difficult to accurately assess using multispectral remote sensing (Furbank et al., 2019). However, the relationship between multispectral VIs and RUE can help in the identification of valuable predictors for future modeling. RECI, NIR/G, NDRE, and SCCCI were the top four VIs in predicting RUE (**Figure 6**). Similarly, the red-edge and green raw bands explained 37% and 34% of the variation in RUE for the validation data set. All the aforementioned VIs and raw reflectance bands have shown to be sensitive to canopy chlorophyll content for crops including corn and cotton (Tumbo et al., 2002; Gitelson et al., 2003a; Bausch et al., 2008; Ritchie et al., 2010; Raper and Varco, 2014; Ballester et al., 2017). The specific absorption coefficient of chlorophyll for the green and red-edge bands is lower than the red and blue bands, making the VIs based on green and red-edge bands sensitive to changes in chlorophyll content in plant tissues (Gitelson and Merzlyak, 1997; Gitelson et al., 2003b). Because the quantum efficiency of primary photochemistry is influenced by canopy chlorophyll content per unit leaf area (Porcar-Castell et al., 2021), the aforementioned VIs may relate to canopy-level RUE. There are limited studies estimating canopy RUE from multispectral VIs. However, Robles-zazueta et al. (2021) showed potential of hyperspectral VIs and partial least square regression (PSLR) modelling to predict RUE in wheat, where EVI and PRI predicted RUE with 70% accuracy.

Additionally, this study showed the potential of using a mechanistic model (RUE = Biomass/IPAR) from estimated IPAR and biomass to derive RUE in cotton. The estimated RUE from the measured biomass and RVI-based IPAR_f_ model was able to explain 84% of measured RUE (**Figure 7**). A similar approach to obtain RUE from measured biomass and remotely sensed light interception has been used in corn (Tewes and Schellberg, 2018) and soybean (Phillips et al., 2020) as well. Further, in this study, actual biomass measurements were replaced with estimates from the MSAVI-based biomass model, which resulted in a reduced prediction accuracy of 18%. The reason for the reduced performance could be due to the error and inaccuracies associated with biomass prediction (Robles-zazueta et al., 2021). As previously discussed, cotton is an indeterminate crop, and its above-ground biomass during the season consists of both vegetative and reproductive structures. Therefore, the spectral VIs may not represent the biomass contributed by the bolls in lower canopy. Moreover, due to cotton’s indeterminate growth pattern, it is challenging to uniformly sample above-ground biomass throughout the season.

### 4.2 Estimation of lint yield and HI

The second objective of this study was to estimate cotton lint yield using cotton fiber index (CFI). CFI explained 69% of the variation in cotton lint yield (**Figure 8**), which is somewhat lower than the result reported by Huang et al. (2016) (R^2 ^= 0.83) and Feng et al. (2020) (R^2 ^= 0.90). However, the performance of CFI in explaining lint yield variation was better than previous studies that used in-season vegetation indices to predict yield (R^2 ^= 0.47 to 0.60) (Gutierrez et al., 2012; Ballester et al., 2017). There are a few limitations of using CFI from UAV-based RGB imagery which could introduce some error and possibly the reason for low accuracy observed in our study. CFI does not consider the cotton bolls present in the lower and middle of the canopy (Feng et al., 2020; Siegfried et al., 2023), and there is a potential for misclassification of soil or background pixels as white cotton bolls. Also, the UAV flight height can have an influence on the image resolution for cotton pixel detection. The lower flight height can increase the detection accuracy but can limit the amount of area covered as well as increase time for image processing. To increase the yield-predicting performance of CFI, future research could focus on identifying low cost remote sensing predictors, such as UAV-based plant height or white flower detection during the season, which can account for the cotton bolls in the middle and below the canopy. Utilizing machine learning and convolutional neural networks to accurately delineate cotton pixels from the surrounding soil pixels could also improve yield prediction (Li F. et al., 2022; Shi et al., 2022).

Finally, the last objective of this study was to investigate the utility of the developed models for lint yield and above-ground biomass in predicting cotton harvest index (HI). No significant relationship was observed between the measured and predicted HI when the data from both years was combined. It was also found that the HI models performed differently for each growing season (**Figure 9**), with the estimated HI explaining 40-49% of the variation in measured HI for the 2022 growing season. The lack of a significant relationship over the two years of the study could be attributed to the combined errors in the lint yield and biomass prediction. As discussed earlier, for an indeterminate crop such as cotton, nadir UAV imagery cannot fully account for the reproductive structure or cotton bolls in the lower and middle canopy that contribute to both above-ground biomass and lint yield (Shi et al., 2022; Siegfried et al., 2023). Furthermore, previous studies have reported varying responses of cotton HI (indifferent to inverse relationship) to different nitrogen levels (Kimball and Mauney, 1993; Gerik et al., 1994; Li et al., 2017), which could be another reason for the varied responses observed in the different seasons.

### 4.3 Model applications, limitations, and implication for future efforts

The models developed to estimate the yield-contributing physiological parameters in cotton showed low to strong performance. The models developed in this study can estimate the yield determining physiological parameters throughout the growing season and are relatively simple with only two required input predictors- VI from multispectral imagery and GDD (growth stage parameter). This makes use of these parameters a viable method to measure the light interception and above-ground biomass rapidly and accurately during the cotton growing season and also expands the potential of these models to be used by researchers and industry in cotton management and breeding. Previous work showed that low nitrogen stress in cotton primarily reduces the light interception of the cotton canopy, resulting in significant yield loss (Pokhrel et al., 2023). The models, therefore, can be applied to measure light interception and identify yield-limiting low nitrogen circumstances in-season. The models have ability to aid in agronomic decision making in order to prevent significant yield loss. Similarly, excessive irrigation can cause excessive above-ground biomass resulting in a lower number of cotton bolls and boll mass, ultimately causing significant yield loss (Ermanis et al., 2020). The models from this study can be potentially applied to regulate the irrigation requirement of a cotton plant during the growing season based on their light interception and biomass gain to avoid yield loss due to excess growth. For cotton breeding, the models can be utilized for high-throughput phenotyping of a large number of cotton genotypes for yield determining physiological parameters without the need for labor-intensive and time-consuming manual measurements. Finally, results attained in this study can be used to expand and improve future research on predicting functional yield drivers of cotton from aerial imagery.

One of the limitations of this study is that the modeling and validation were limited to data collected within 372 GDD to 1253 GDD, with no extrapolation, and it would be beneficial to test these models outside this range in future studies. Furthermore, the models were developed based on a two-year study of a single cotton cultivar’s response to five different nitrogen application rates at an experimental site. Inclusion of training data from different cotton cultivars across multiple production environments can enhance model performance. It is suggested that future research should focus on increasing the model’s robustness for its transferability across a wide range of genotypes and environmental conditions. Independent validation from different locations would also help in determining the model’s prediction ability. In the future, more robust models can be created by exposing the crop to a broader range of growth and yield-altering factors. Additionally, due to the limited number of data points, validation for the lint yield modeling was not performed. Validation for the use of CFI in lint yield prediction can be done in the future. There is also the possibility of misclassification while separating cotton white pixels and background soils for CFI calculation. Future research can work on assessing quality of those filtering techniques to avoid the misclassifications. Moreover, top-view UAV imagery from 45 m height may not fully capture the cotton bolls present in the middle section of cotton plants. Future research can investigate the influence of different flight parameters such as flight height and sensor resolution on accurate estimation of cotton lint yield and above-ground biomass prediction. This would potentially aid in increasing the estimation accuracy of the cotton harvest index. Overall, this study is the first attempt of its kind to use low-cost UAV RGB and multispectral imagery to predict yield-determining physiological parameters of cotton throughout the season and the results obtained here shows a strong potential to utilize and expand the use of remotely-sensed imagery for estimating yield-driving functional traits of cotton.

## 5 Conclusions

The objectives of this study were to predict IPAR_f_, RUE, and biomass of cotton during the growing season using VIs derived from UAV-based multispectral imagery and GDD, and to estimate cotton HI from biomass models and CFI-based lint yield estimates. Estimated IPAR_f_ using models based on RVI, RECI, NDRE and SCCCI showed strong relationships with actual IPAR_f_ values during cross-validation. RUE was best explained by VIs that used red-edge, green, and near-infrared bands such as RECI, NIR/G, NDRE, and SCCCI, which are linked to the chlorophyll content per unit leaf area in past studies. Models based on MSAVI, OSAVI, RVI, and SAVI explained most of the variation in above-ground biomass during cross-validation. CFI had a moderate relationship with the machine-harvested lint yields. Estimated HI based on CFI-based lint yield estimates and biomass models showed no significant relationships with actual HI values and only weak relationships with actual values during the 2022 growing season. Further research towards accurate estimation of lint yield and biomass is recommended to predict cotton harvest index. Thus, we can conclude that UAV-based RGB and multispectral imagery can be utilized to predict some yield-determining physiological parameters in cotton.

## Data availability statement

The raw data supporting the conclusions of this article will be made available by the authors, without undue reservation.

## Author contributions

Conceptualization: JS, SV, and AP. Data curation: SV and AP. Formal analysis: AP and SV. Funding acquisition: JS and HS. Investigation: AP, SV, VP, DC, JL, and CB. Methodology: SV, JS and AP. Project administration: SV, JS, and AP. Resources: SV, JS and HS. Writing-Original draft: AP, SV, and JS. Writing-review and editing: SV, JS, AP, GV, LH, VP, DC, JL, and CB. All authors contributed to the article and approved the submitted version.

## Glossary

**Table:**

AICc	Akaike Information Criterion Value
BIC	Bayesian Information Criterion
CFI	Cotton Fiber Index
EVI	Enhanced Vegetation Index
EVI2	Enhanced Vegetation Index 2
ExG	Excessive Greenness Index
GCPs	Ground Control Points
GDDs	Growing Degree Days
GNDVI	Green Normalized Difference Vegetation Index
GNSS	Global Navigation Satellite System
GPS	Global Positioning System
GRVI	Green Red Vegetation Index
HI	Harvest Index
IPAR	Intercepted Photosynthetically Active Radiation
IPAR_f_	Fraction of Intercepted Photosynthetically Active Radiation
LAI	Leaf Area Index
MSAVI	Modified Soil Adjusted Vegetation Index
N	Nitrogen
NDRE	Normalized Difference Red Edge Index
NDVI	Normalized Difference Vegetation Index
NIR	Near infrared
NIR/G	Near infrared to Green ratio
OSAVI	Optimized Soil Adjusted Vegetation Index
PAR	Photosynthetically Active Radiation
PRI	Photochemical Reflectance Index
R^2^	Coefficient of Determination
RE/R	Redegde to Red ratio
RECI	Rededge Chlorophyll Index
RGB	Red Green Blue Composite
RGBVI	Red Green Blue Vegetation Index
RMSE	Root Mean Sun Square Error
ROI	Region of interest
RTK	Real Time Kinematics
RUE	Radiation Use Efficiency
RVI	Ratio Vegetation Index
SAVI	Soil Adjusted Vegetation Index
SCCCI	Simplified Canopy Chlorophyll Content Index
SIF	Sun-induced Fluorescence
TCARI	Transformed Chlorophyll Absorption Reflectance Index
UAV	Unmanned Aerial Vehicle
VARI	Visible Atmospherically Resistant Index
VIs	Vegetation Indices
WDRVI	Wide Dynamic Range Vegetation Index

## References

[B1] Adams C. B. Ritchie G. L. Rajan N. (2021). Cotton phenotyping and physiology monitoring with a proximal remote sensing system. Crop Sci. 61 (2), 1317–1327. doi: 10.1002/csc2.20434

[B2] Ashapure A. Jung J. Chang A. Oh S. Maeda M. Landivar J. (2019). A comparative study of RGB and multispectral sensor-based cotton canopy cover modelling using multi-temporal UAS data. Remote Sens. 11 (23). doi: 10.3390/rs11232757

[B3] Ballester C. Jiménez-Bello M. A. Castel J. R. Intrigliolo D. S. (2013). Usefulness of thermography for plant water stress detection in citrus and persimmon trees. Agric. For. Meteorol. 168, 120–129. doi: 10.1016/j.agrformet.2012.08.005

[B4] Ballester C. Hornbuckle J. Brinkhoff J. Smith J. Quayle W. (2017). Assessment of in-season cotton nitrogen status and lint yield prediction from unmanned aerial system imagery. Remote Sens. 9 (11), 1149–1167. doi: 10.3390/rs9111149

[B5] Bange M. P. Milroy S. P. (2000). “Assessing effects of canopy nitrogen and light distribution on radiation use efficiency of cotton,” in New frontiers in cotton research. Proceedings of the Second World Cotton Conference, Athens Greece (Thessaloniki, Greece: P. Petridis Publishers. Athens, Greece), 498–501.

[B6] Barbedo J. G. A. (2019). A review on the use of unmanned aerial vehicles and imaging sensors for monitoring and assessing plant stresses. Drones 3 (2), 1–27. doi: 10.3390/drones3020040

[B7] Bausch W. C. Halvorson A. D. Cipra J. (2008). Quickbird satellite and ground-based multispectral data correlations with agronomic parameters of irrigated maize grown in small plots. Biosyst. Eng. 101 (3), 306–315. doi: 10.1016/j.biosystemseng.2008.09.011

[B8] Bendig J. Yu K. Aasen H. Bolten A. Bennertz S. Broscheit J.. (2015). Combining UAV-based plant height from crop surface models, visible, and near infrared vegetation indices for biomass monitoring in barley. Int. J. Appl. Earth Observ. Geoinform. 39, 79–87. doi: 10.1016/j.jag.2015.02.012

[B9] Bhattacharya A. (2019). “Radiation-Use efficiency under different climatic conditions,” in Changing Climate and Resource Use Efficiency in Plants (Academic Press in UK and USA: Elsevier), 51–109. doi: 10.1016/b978-0-12-816209-5.00002-7

[B10] Boegh E. Soegaard H. Broge N. Schelde K. Thomsen A. Hasager C. B.. (2002). Airborne multispectral data for quantifying leaf area index, nitrogen concentration, and photosynthetic efficiency in agriculture. Remote Sens. Environ. 81 (2–3), 179–193. doi: 10.1016/S0034-4257(01)00342-X

[B11] Bondada B. R. Oosterhuis D. M. (2001). Canopy photosynthesis, specific leaf weight, and yield components of cotton under varying nitrogen supply. J. Plant Nutr. 24 (3), 469–477. doi: 10.1081/PLN-100104973

[B12] Brandão Z. N. Sofiatti V. Bezerra J. R. C. Ferreira G. B. Medeiros J. C. (2015). Spectral reflectance for growth and yield assessment of irrigated cotton. Aust. J. Crop Sci. 9 (1), 75–84.

[B13] Brodrick R. Bange M. P. Milroy S. P. Hammer G. L. (2013). Physiological determinants of high yielding ultra-narrow row cotton: Canopy development and radiation use efficiency. Field Crops Res. 148, 86–94. doi: 10.1016/j.fcr.2012.05.008

[B14] Campoy J. Campos I. Plaza C. Calera M. Jiménez N. Bodas V.. (2019). Water use efficiency and light use efficiency in garlic using a remote sensing- based approach. Agric. Water Manage. 219, 40–48. doi: 10.1016/j.agwat.2019.03.032

[B15] Chao Z. Liu N. Zhang P. Ying T. Song K. (2019). Estimation methods developing with remote sensing information for energy crop biomass: A comparative review. Biomass Bioenergy 122 (February), 414–425. doi: 10.1016/j.biombioe.2019.02.002

[B16] Chen P. (2019). “‘Cotton leaf area index estimation using unmanned aerial vehicle multi-spectral images’,” in IGARSS 2019-2019 IEEE International Geoscience and Remote Sensing Symposium. IEEE. 6251–6254.

[B17] Chen P. Wang F. (2020). New textural indicators for assessing above-ground cotton biomass extracted from optical imagery obtained via unmanned aerial vehicle. Remote Sens. 12 (24). doi: 10.3390/rs12244170

[B18] Cheng Y. Middleton E. M. Zhang Q. Huemmrich K. F. Campbell P. K. E. Corp L. A.. (2013). Integrating solar induced fluorescence and the photochemical reflectance index for estimating gross primary production in a cornfield. Remote Sens. 5, 6857–6879. doi: 10.3390/rs5126857

[B19] Clevers J. G. P. W. Leeuwen H. J. C. V. Verhoef W. (1989). Estimating APAR by means of vegetation indices: A sesitivity analysis. The Netherlands.

[B20] Conaty W. C. Constable G. A. (2020). ‘Factors responsible for yield improvement in new Gossypium hirsutum L. cotton cultivars. Field Crops Res. 250 (May 2019), 107780. doi: 10.1016/j.fcr.2020.107780

[B21] Deng L. Mao Z. Li X. Hu Z. Duan F. Yan Y. (2018). UAV-based multispectral remote sensing for precision agriculture: A comparison between different cameras. ISPRS J. Photogramm. Remote Sens. 146, 124–136. doi: 10.1016/j.isprsjprs.2018.09.008

[B22] Dunn P. K. Smyth G. K. (2018). Generalized Linear Models with Examples in R. Eds. DeVeaux R. Fienberg S. E. Olkin I. (New York, NY: Springer US). doi: 10.1007/978-1-4419-0118-7

[B23] Ermanis A. Gobbo S. Snider J. L. Cohen Y. Liakos V. Lacerda L.. (2020). Defining physiological contributions to yield loss in response to irrigation in cotton. J. Agron. Crop Sci. 207 (2), 186–196. doi: 10.1111/jac.12453

[B24] Falkowski M. J. Gessler P. E. Morgan P. Hudak A. T. Smith A. M. S. (2005). Characterizing and mapping forest fire fuels using ASTER imagery and gradient modeling. For. Ecol. Manage. 217 (2–3), 129–146. doi: 10.1016/j.foreco.2005.06.013

[B25] Feng A. Zhou J. Vories E. D. Sudduth K. A. Zhang M. (2020). Yield estimation in cotton using UAV-based multi-sensor imagery. Biosyst. Eng. 193, 101–114. doi: 10.1016/j.biosystemseng.2020.02.014

[B26] Fu P. Montes C. M. Siebers M. H. Gomez-casanovas N. McGrath J. M. Ainsworth E. A.. (2022). Advances in field-based high-throughput photosynthetic phenotyping. J. Exp. Bot. 73 (10), 3157–3172. doi: 10.1093/jxb/erac077 35218184 PMC9126737

[B27] Furbank R. T. Jimenez-Berni J. A. George-Jaeggli B. Potgieter A. B. Deery D. M. (2019). Field crop phenomics: enabling breeding for radiation use efficiency and biomass in cereal crops. New Phytol. 223 (4), 1714–1727. doi: 10.1111/nph.15817 30937909

[B28] Garbulsky M. F. Peñuelas J. Gamon J. Inoue Y. Filella I. (2011). The photochemical re fl ectance index (PRI) and the remote sensing of leaf, canopy and ecosystem radiation use efficiencies A review and meta-analysis. Remote Sens. Environ. 115, 281–297. doi: 10.1016/j.rse.2010.08.023

[B29] Gerik T. J. Jackson B. S. Stockle C. O. Rosenthal W. D. (1994). Plant nitrogen status and boll load of cotton. Agron. J. 86 (3), 514–518. doi: 10.2134/agronj1994.00021962008600030011x

[B30] Gitelson A. A. (2004). Wide dynamic range vegetation index for remote quantification of biophysical characteristics of vegetation. J. Plant Physiol. 161 (2), 165–173. doi: 10.1078/0176-1617-01176 15022830

[B31] Gitelson A. A. Kaufman Y. J. Stark R. Rundquist D. (2002). Novel algorithms for remote estimation of vegetation fraction. Remote Sens. Environ. 80 (1), 76–87. doi: 10.1016/S0034-4257(01)00289-9

[B32] Gitelson A. A. Gritz Y. Merzlyak M. N. (2003a). Relationships between leaf chlorophyll content and spectral reflectance and algorithms for non-destructive chlorophyll assessment in higher plant leaves. J Plant Physiol. 160, 271–282. doi: 10.1078/0176-1617-00887 12749084

[B33] Gitelson A. A. Gritz Y. Merzlyak M. N. (2003b). Relationships between leaf chlorophyll content and spectral reflectance and algorithms for non-destructive chlorophyll assessment in higher plant leaves. J. Plant Physiol. 160 (3), 271–282. doi: 10.1078/0176-1617-00887 12749084

[B34] Gitelson A. A. Merzlyak M. N. (1997). Remote estimation of chlorophyll content in higher plant leaves. Int. J. Remote Sens. 18 (12), 2691–2697. doi: 10.1080/014311697217558

[B35] Gonzalez-Dugo V. Zarco-Tejada P. Nicolás E. Nortes P. A. Alarcón J. J. Intrigliolo D. S.. (2013). Using high resolution UAV thermal imagery to assess the variability in the water status of five fruit tree species within a commercial orchard. Precis. Agric. 14 (6), 660–678. doi: 10.1007/s11119-013-9322-9

[B36] Guillen-Climent M. L. Zarco-tejada P. J. Berni J. A. J. North P. R. J. Villalobos F. J. (2012). Mapping radiation interception in row-structured orchards using 3D simulation and high-resolution airborne imagery acquired from a UAV. Precis. Agric. 13, 473–500. doi: 10.1007/s11119-012-9263-8

[B37] Gutierrez M. Norton R. Thorp K. R. Wang G. (2012). Association of spectral reflectance indices with plant growth and lint yield in upland cotton. Crop Sci. 52 (2), 849–857. doi: 10.2135/cropsci2011.04.0222

[B38] Haboudane D. Miller J. R. Tremblay N. Zarco-Tejada P. J. Dextraze L. (2002). Integrated narrow-band vegetation indices for prediction of crop chlorophyll content for application to precision agriculture. Remote Sens. Environ. 81 (2–3), 416–426. doi: 10.1016/S0034-4257(02)00018-4

[B39] Hand C. Culpepper S. Harris G. Kemerait B. Liu Y. Perry C.. (2022) ‘2022 GEORGIA Cotton Production Guide’. Available at: https://secure.caes.uga.edu/extension/publications/files/pdf/AP124-2_1.PDF.

[B40] Hatfield J. L. Asrar G. Kanemasu E. T. (1984). Intercepted photosynthetically active radiation estimated by spectral reflectance. Remote Sens. Environ. 14 (1–3), 65–75. doi: 10.1016/0034-4257(84)90008-7

[B41] Hatfield J. L. Prueger J. H. (2010). Value of using different vegetative indices to quantify agricultural crop characteristics at different growth stages under varying management practices. Remote Sens. 2 (2), 562–578. doi: 10.3390/rs2020562

[B42] Hilker T. Coops N. C. Wulder M. A. Black T. A. Guy R. D. (2008). The use of remote sensing in light use efficiency based models of gross primary production: A review of current status and future requirements. Sci. Total Environ. 404, 411–423. doi: 10.1016/j.scitotenv.2007.11.007 18063011

[B43] Huang Y. B. Sui R. X. Thomson S. J. Fisher D. K. (2013). Estimation of cotton yield with varied irrigation and nitrogen treatments using aerial multispectral imagery. Int. J. Agric. Biol. Eng. 6 (2), 37–41. doi: 10.3965/j.ijabe.20130602.00?

[B44] Huang Y. Brand H. J. Sui R. Thomson S. J. Furukawa T. Ebelhar M. W. (2016). Cotton yield estimation using very high-resolution digital images acquired with a low-cost small unmanned aerial vehicle. Trans. ASABE 59 (6), 1563–1574. doi: 10.13031/trans.59.11831

[B45] Huete A. R. (1988). A soil-adjusted vegetation index (SAVI). Remote Sens. Environ. 25 (3), 295–309. doi: 10.1016/0034-4257(88)90106-X

[B46] Huete A. Didan K. Miura T. Rodriguez E. P. Gao X. Ferreira L. G. (2002). Overview of the radiometric and biophysical performance of the MODIS vegetation indices. Remote Sens. Enviroment 83, 195–213. doi: 10.1016/S0034-4257(02)00096-2

[B47] Hunsaker D. J. Pinter P. J. Barnes E. M. Kimball B. A. (2003). Estimating cotton evapotranspiration crop coefficients with a multispectral vegetation index. Irrigation Sci. 22 (2), 95–104. doi: 10.1007/s00271-003-0074-6

[B48] Jackson R. D. (1986). Remote sensing of biotic and abiotic plant stress. Annu. Rev. Phytopathol. 24, 265–287. doi: 10.1146/annurev.py.24.090186.001405

[B49] Jafarbiglu H. Pourreza A. (2022). A comprehensive review of remote sensing platforms, sensors, and applications in nut crops. Comput. Electron. Agric. 197 (July 2021), 106844. doi: 10.1016/j.compag.2022.106844

[B50] Jiang Z. Huete A. R. Didan K. Miura T. (2008). Development of a two-band enhanced vegetation index without a blue band. Remote Sens. Environ. 112 (10), 3833–3845. doi: 10.1016/j.rse.2008.06.006

[B51] Junhua B. Shaokun L. Keru W. Xueyan S. Bing C. (n.d.). Estimation models of cotton aboveground fresh biomass based on field hyperspectral remote sensing, 311–316. Available at: http://europepmc.org/abstract/CBA/636815.

[B52] Kimball B. A. Mauney J. R. (1993). Response of cotton to varying CO2, irrigation, and nitrogen: yield and growth. Agron. J. 85 (3), 706–712. doi: 10.2134/agronj1993.00021962008500030035x

[B53] Kumar L. Schmidt K. Dury S. Skidmore A. (2002). “Imaging spectrometry and vegetation science,” in Imaging Spectrometry. Remote Sensing and Digital Image Processing. Ed. Meer F.D., J. S. M. D. (Springer), 111–155.

[B54] Li C. Knowlton A. Brown S. Ritchie G. (2011). A comparative study of a microgin with a lab gin stand and commercial gins in southeast United States. Appl. Eng. Agric. 27 (2), 167–176. doi: 10.13031/2013.36488

[B55] Li P. Dong H. Zheng C. Sun M. Liu A. Wang G.. (2017). Optimizing nitrogen application rate and plant density for improving cotton yield and nitrogen use efficiency in the North China Plain. PloS One 12 (10), 1–15. doi: 10.1371/journal.pone.0185550 PMC5628833 28981538

[B56] Li B. Xu X. Han J. Zhang L. Bian C. Jin L.. (2019). The estimation of crop emergence in potatoes by UAV RGB imagery. Plant Methods 15 (1), 1–13. doi: 10.1186/s13007-019-0399-7 30792752 PMC6371461

[B57] Li F. Bai J. Zhang M. Zhang R. (2022). Yield estimation of high-density cotton fields using low-altitude UAV imaging and deep learning. Plant Methods 18 (1), 1–11. doi: 10.1186/s13007-022-00881-3 35477580 PMC9044671

[B58] Li Z. Zhao Y. Taylor J. Gaulton R. Jin X. Song X.. (2022). Comparison and transferability of thermal, temporal and phenological-based in-season predictions of above-ground biomass in wheat crops from proximal crop reflectance data. Remote Sens. Environ. 273, 112967. doi: 10.1016/j.rse.2022.112967

[B59] Lu S. (2022) Market Size of the Global Textile and Apparel Industry: 2016 to 2021/2022, FASH455 Global Apparel & Textile Trade and Sourcing. Available at: https://shenglufashion.com/2018/12/18/market-size-of-the-global-textile-and-apparel-industry-2016-to-2021-2022/ (Accessed 11 September 2022).

[B60] Lv Z. Meng R. Man J. Zeng L. Wang M. Xu B. (2021). Modeling of winter wheat fAPAR by integrating Unmanned Aircraft Vehicle-based optical, structural and thermal measurement. Int. J. Appl. Earth Observ. Geoinform. 102, 102407. doi: 10.1016/j.jag.2021.102407

[B61] Merrick T. Jorge M. L. S. P. Silva T. S. F. Pau S. Broadbent E. N. Bennartz R.. (2020). Characterization of chlorophyll fluorescence, absorbed photosynthetically active radiation, and reflectance-based vegetation index spectroradiometer measurements. Int. J. Remote Sens. 41 (17), 6755–6782. doi: 10.1080/01431161.2020.1750731

[B62] Moghimi A. Yang C. Miller M. E. Kianian S. F. Marchetto P. M. (2018). A novel approach to assess salt stress tolerance in wheat using hyperspectral imaging. Front. Plant Sci. 9, 1–17. doi: 10.3389/fpls.2018.01182 30197650 PMC6117507

[B63] Monteith J. L. (1972). Solar radiation and productivity in tropical ecosystems. J. Appl. Ecol. 9 (3), 747–766. doi: 10.2307/2401901

[B64] Nelder J. A. Wedderburn R. W. M. (1972). Generalized linear models. J. R. Stat. Soc. 135 (3), 370–384. doi: 10.1016/B978-0-08-044894-7.01331-2

[B65] Parkash V. Snider J. L. Sintim H. Y. Hand L. C. Virk G. Pokhrel A. (2023). Differential sensitivities of photosynthetic processes and carbon loss mechanisms govern N-induced variation in net carbon assimilation rate for field-grown cotton. J. Exp. Bot. 74 (8), 2638–2652. doi: 10.1093/jxb/erad038 36715336

[B66] Pellegrini P. Sadras V. O. Oesterheld M. Bella C. M. Piñeiro G.. (2020). Simple regression models to estimate light interception in wheat crops with Sentinel-2 and a handheld sensor. Crop Sci. 60, 1607–1616. doi: 10.1002/csc2.20129

[B67] Phillips X. A. Kandel Y. R. Licht M. A. Mueller D. S. (2020). Estimating soybean radiation use efficiency using a UAV in Iowa. Agronomy 10 (2002), 1–13. doi: 10.3390/agronomy10122002

[B68] Pinter P. J. Hatfield J. L. Schepers J. S. Barnes E. M. Moran M. S. Daughtry C. S.T.. (2003). Remote sensing for crop management. Photogramm. Eng. Remote Sens. 69 (6), 647–664. doi: 10.14358/PERS.69.6.647

[B69] Pokhrel A. Snider J. L. Virk S. Sintim H. Y. Hand L. C. Vellidis G.. (2023). Quantifying physiological contributions to nitrogen-induced yield variation in field-grown cotton. Field Crops Res. 299. doi: 10.1016/j.fcr.2023.108976

[B70] Porcar-Castell A. Malenovský Z. Magney T. Van Wittenberghe S. Fernández-Marín B. Maignan F.. (2021). Chlorophyll a fluorescence illuminates a path connecting plant molecular biology to Earth-system science. Nat. Plants 7 (8), 998–1009. doi: 10.1038/s41477-021-00980-4 34373605

[B71] Prasad N. R. Patel N. R. Danodia A. Manjunath K. R. (2021). Comparative performance of semi − empirical based remote sensing and crop simulation model for cotton yield prediction. Model. Earth Syst. Environ. doi: 10.1007/s40808-021-01180-x

[B72] Qi J. Chehbouni A. Huete A. R. Kerr Y. H. Sorooshian S. (1994). A modified soil adjusted vegetation index. Remote Sens. Environ. 48 (2), 119–126. doi: 10.1016/0034-4257(94)90134-1

[B73] Raper T. B. Varco J. J. (2014). Canopy-scale wavelength and vegetative index sensitivities to cotton growth parameters and nitrogen status. Precis. Agric. 16 (1), 62–76. doi: 10.1007/s11119-014-9383-4

[B74] Ritchie G. L. Bednarz C. W. Jost P. H. Brown S. M. (2004). Cotton growth and development (Georgia, United States: University of Georgia Cooperative Extension Service Tifton, GA). doi: 10.32473/edis-ag235-2005

[B75] Ritchie G. L. Sullivan D. G. Vencill W. K. Bednarz C. W. Hook J. E. (2010). Sensitivities of norMalized difference vegetation index and a green/red Ratio index to cotton ground cover fraction. Crop Sci. 50 (3), 1000–1010. doi: 10.2135/cropsci2009.04.0203

[B76] Robles-zazueta C. A. Molero G. Pinto F. Foulkes M. J. (2021). Field-based remote sensing models predict radiation use efficiency in wheat. J. Exp. Bot. 72 (10), 3756–3773. doi: 10.1093/jxb/erab115 33713415 PMC8096595

[B77] Rondeaux G. Steven M. Baret F. (1996). Optimization of soil-adjusted vegetation indices. Remote Sens. Environ. 55 (2), 95–107. doi: 10.1016/0034-4257(95)00186-7

[B78] Rouse J. W. (1974). Monitoring the vernal advancement and retrogradation (green wave effect) of natural vegetation (NTRS - NASA Technical Reports Server).

[B79] Sankaran S. Khot L. R. Espinoza C. Z. Jarolmasjed S. Sathuvalli V. R. Vandemark G. J.. (2015). Low-altitude, high-resolution aerial imaging systems for row and field crop phenotyping: A review. Eur. J. Agron. 70, 112–123. doi: 10.1016/j.eja.2015.07.004

[B80] Sharma A. Deepa R. Sankar S. Pryor M. Stewart B. Johnson E.. (2021). Use of growing degree indicator for developing adaptive responses: A case study of cotton in Florida. Ecol. Indic. 124, 107383. doi: 10.1016/j.ecolind.2021.107383

[B81] Shi G. Du X. Du M. Li Q. Tian X. Ren Y.. (2022). Cotton yield estimation using the remotely sensed cotton boll index from UAV images. Drones 6 (9). doi: 10.3390/drones6090254

[B82] Siegfried J. Adams C. B. Rajan N. Hague S. Schnell R. Hardin R. (2023). Combining a cotton “Boll Area Index” with in-season unmanned aerial multispectral and thermal imagery for yield estimation. Field Crops Res. 291. doi: 10.1016/j.fcr.2022.108765

[B83] Snider J. Bange M. Heitholt J. (2021b). “Cotton,” in Crop Physiology Case Histories for Major Crops. Eds. Sadras V. O. Calderini D. F. (UK and USA: Academic Press), 714–746. doi: 10.1016/B978-0-12-819194-1.00022-0

[B84] Snider J. Harris G. Roberts P. Meeks C. Chastain D. Bange M.. (2021a). Cotton physiological and agronomic response to nitrogen application rate. Field Crops Res. 270. doi: 10.1016/j.fcr.2021.108194

[B85] Soil Survey Staff, Natural Resources Conservation Service and United States Department of Agriculture Web soil survey. Available at: https://websoilsurvey.sc.egov.usda.gov/App/WebSoilSurvey.aspx (Accessed 20 January 2023). (no date).

[B86] Sripada R. P. Heiniger R. W. White J. G. Weisz R. (2005). Aerial color infrared photography for determining late-season nitrogen requirements in corn. Agron. J. 97 (5), 1443–1451. doi: 10.2134/agronj2004.0314

[B87] Sui R. Byler R. K. Delhom C. D. (2017). Effect of nitrogen application rates on yield and quality in irrigated and rainfed cotton. J. Cotton Sci. 21 (2), 113–121. doi: 10.56454/XZQP5457

[B88] Sun S. Li C. Paterson A. H. Jiang Y. Xu R. Robertson J. S.. (2018). In-field high throughput phenotyping and cotton plant growth analysis using LiDAR. Front. Plant Sci. 9, 1–17. doi: 10.3389/fpls.2018.00016 29403522 PMC5786533

[B89] Tan C. Samanta A. Jin X. Tong L. Ma C. Knyazikhin Y.. (2013). Using hyperspectral vegetation indices to estimate the fraction of photosynthetically active radiation absorbed by corn canopies. Int. J. Remote Sens. 34 (24), 8789–8802. doi: 10.1080/01431161.2013.853143

[B90] Tan C. Wang D. Zhou J. Du Y. Luo M. Zhang Y. (2018). Remotely assessing fraction of Photosynthetically Active Radiation (fPAR) for wheat canopies based on hyperspectral vegetation indexes. Front. Plant Sci. 9, 1–9. doi: 10.3389/fpls.2018.00776 29930568 PMC5999760

[B91] Tewes A. Schellberg J. (2018). Towards remote estimation of radiation use efficiency in maize using UAV-based low-cost camera imagery. Agronomy 8 (1), 1–15. doi: 10.3390/agronomy8020016

[B92] Tucker C. J. (1979). Red and photographic infrared linear combinations for monitoring vegetation. Remote Sens. Environ. 8 (2), 127–150. doi: 10.1016/0034-4257(79)90013-0

[B93] Tumbo S. D. Wagner D. G. Heinemann P. H. (2002). ‘Hyperspectral characteristics of corn plants under different chlorophyll levels’. Trans. ASAEE 45 (3), 815–823.

[B94] USDA (2022). Cotton: World Markets and Trade. USDA.

[B95] USDA (2023). Cotton and Wool Outlook: February 2023 (USDA).

[B96] USDA Agricultural Marketing Service (2020) Varieties Planted 2020 Crop. Available at: https://www.ams.usda.gov/mnreports/cnavar.pdf.

[B97] Vatter T. Gracia‐Romero A. Kefauver S. C. Nieto‐Taladriz M. T. Aparicio N. Araus J. L. (2021). Preharvest phenotypic prediction of grain quality and yield of durum wheat using multispectral imaging. Plant J. 109 (6), 1507–1518. doi: 10.1111/tpj.15648 34951491

[B98] Voora V. Larrea C. Bermudez S. (2020). Global Market Report: Cotton, Sustainable Commodities Marketplace Series 2019. Canada.

[B99] Wajid A. Ahmad A. Khaliq T. Alam S. Hussain A. Hussain K.. (2010). Quantification of growth, yield and radiation use efficiency of promising cotton cultivars at varying nitrogen levels. Pakistan J. Bot. 42 (3), 1703–1711.

[B100] Wang T. Liu Y. Wang M. Fan Q. Tian H. Qiao X.. (2021). Applications of UAS in crop biomass monitoring: A Review. Front. Plant Sci. 12, 1–16. doi: 10.3389/fpls.2021.616689 PMC8062761 33897719

[B101] Wiegand C. L. Richardson A. J. Escobar D. E. Gerbermann A. H. (1991). Vegetation indices in crop assessments. Remote Sens. Environ. 35 (2–3), 105–119. doi: 10.1016/0034-4257(91)90004-P

[B102] Woebbecke D. M. Meyer G. E. Von Bargen K. Mortensen D. A. (1995). Color indices for weed identification under various soil, residue, and lighting conditions. Trans. ASAE 38 (1), 259–269. doi: 10.13031/2013.27838

[B103] Wullschleger S. D. Oosterhuis D. M. (1990). Canopy development and photosynthesis of cotton as influenced by nitrogen nutrition. J. Plant Nutr. 13 (9), 1141–1154. doi: 10.1080/01904169009364140

[B104] Xu R. Li C. Paterson A. H. (2019). Multispectral imaging and unmanned aerial systems for cotton plant phenotyping. PloS One 14 (2), e0205083. doi: 10.1371/journal.pone.0205083 30811435 PMC6392284

[B105] Yang C. Bradford J. M. Wiegand C. L. (2001). Airborne multispectral imagery for mapping variable growing conditions and yields of cotton, grain sorghum, and corn. Trans. ASABE 44 (6), 1983–1994.

[B106] Yue J. Yang G. Tian Q. Feng H. Xu K. Zhou C. (2019). Estimate of winter-wheat above-ground biomass based on UAV ultrahigh- ground-resolution image textures and vegetation indices. ISPRS J. Photogramm. Remote Sens. 150, 226–244. doi: 10.1016/j.isprsjprs.2019.02.022

[B107] Zarco-Tejada P. J. Ustin S. L. Whiting M. L. Zarco‐Tejada P. J. Ustin S. L. Whiting M. L. (2005). Temporal and spatial relationships between within-field yield variability in cotton and high-spatial hyperspectral remote sensing imagery. Agron. J. 97 (3), 641–653. doi: 10.2134/agronj2003.0257

[B108] Zhang C. Filella I. Garbulsky M. F. Penuelas J. (2016). Affecting factors and recent improvements of the Photochemical Reflectance Index (PRI) for remotely sensing foliar, canopy and ecosystemic radiation-use efficiences. Remote Sens. 8. doi: 10.3390/rs8090677

[B109] Zhang Y. J. Hou M. Y. Xue H. Y. Liu L. T. Sun H. C. Li C. D.. (2018). Photochemical reflectance index and solar-induced fluorescence for assessing cotton photosynthesis under water-deficit stress. Biol. Plantarum 62 (4), 817–825. doi: 10.1007/s10535-018-0821-4

[B110] Zhao D. Huang L. Li J. Qi J. (2007a). A comparative analysis of broadband and narrowband derived vegetation indices in predicting LAI and CCD of a cotton canopy. ISPRS J. Photogramm. Remote Sens. 62 (1), 25–33. doi: 10.1016/j.isprsjprs.2007.01.003

[B111] Zhao D. Reddy K. R. Kakani V. G. Read J. J. Koti S. (2007b). Canopy reflectance in cotton for growth assessment and lint yield prediction. Eur. J. Agron. 26 (3), 335–344. doi: 10.1016/j.eja.2006.12.001

[B112] Zhao D. H. Li J. L. Qi J. G. (2005). Identification of red and NIR spectral regions and vegetative indices for discrimination of cotton nitrogen stress and growth stage. Comput. Electron. Agric. 48 (2), 155–169. doi: 10.1016/j.compag.2005.03.003

