# Supplementary material for: Estimating yield-contributing physiological parameters of cotton using UAV-based imagery
Source: Front Plant Sci. 2023 Sep 19;14:1248152. doi: 10.3389/fpls.2023.1248152 (PMC10546020; doi:10.3389/fpls.2023.1248152)
Supplement: Supplementary file 1 [file DataSheet_1.pdf]

Supplementary Material

Predicting Yield-Contributing Physiological Parameters of Cotton Using UAV-Based Imagery

Amrit Pokhrel*, Simerjeet Virk, John L. Snider, George Vellidis, Lavesta C. Hand, Henry Y. Sintim, Ved Parkash, Devendra P. Chalise, Joshua M. Lee, and Coleman Byers

***Correspondence:** Corresponding Author: amritpokhrel@uga.edu

1 Supplementary Figures and Tables

1.1 Supplementary Figures

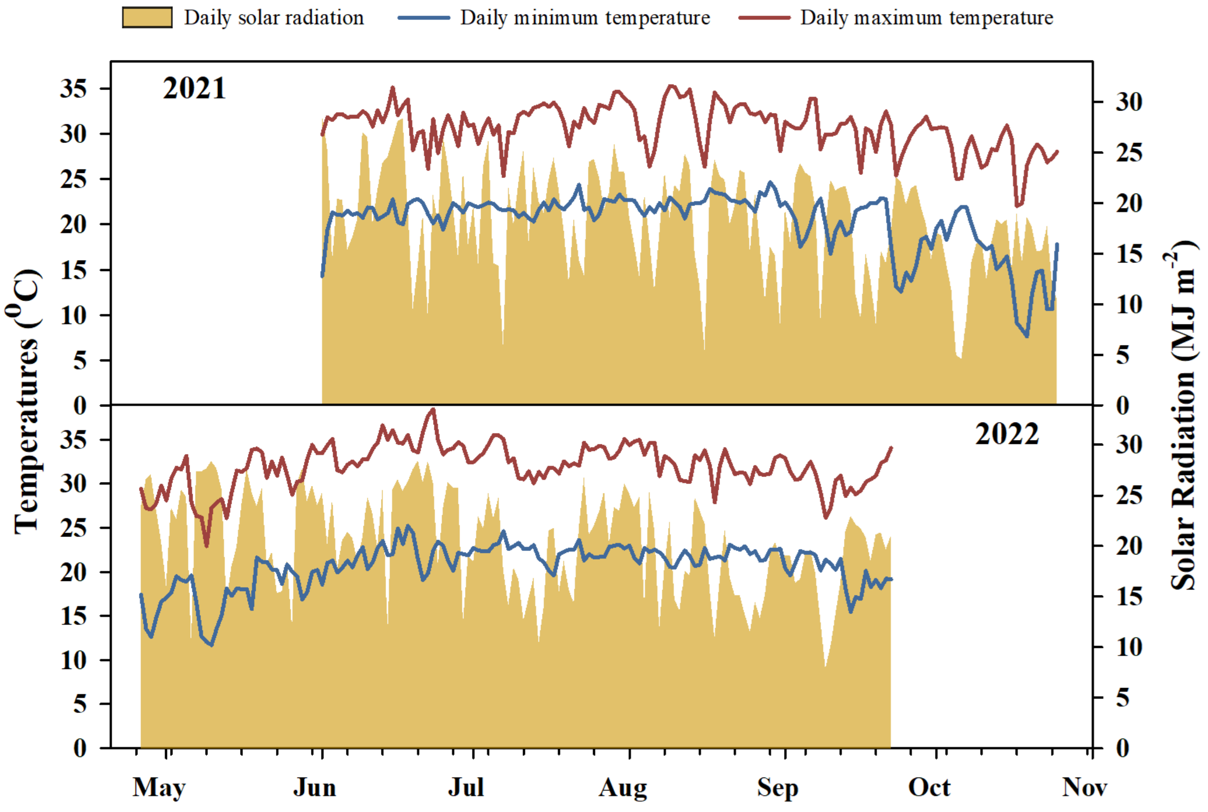

Supplementary Figure 1. Daily solar radiation, daily minimum temperature, and daily maximum temperature from planting to harvest dates at the study sites in Tifton, Georgia, USA during the 2021 and 2022 growing seasons.

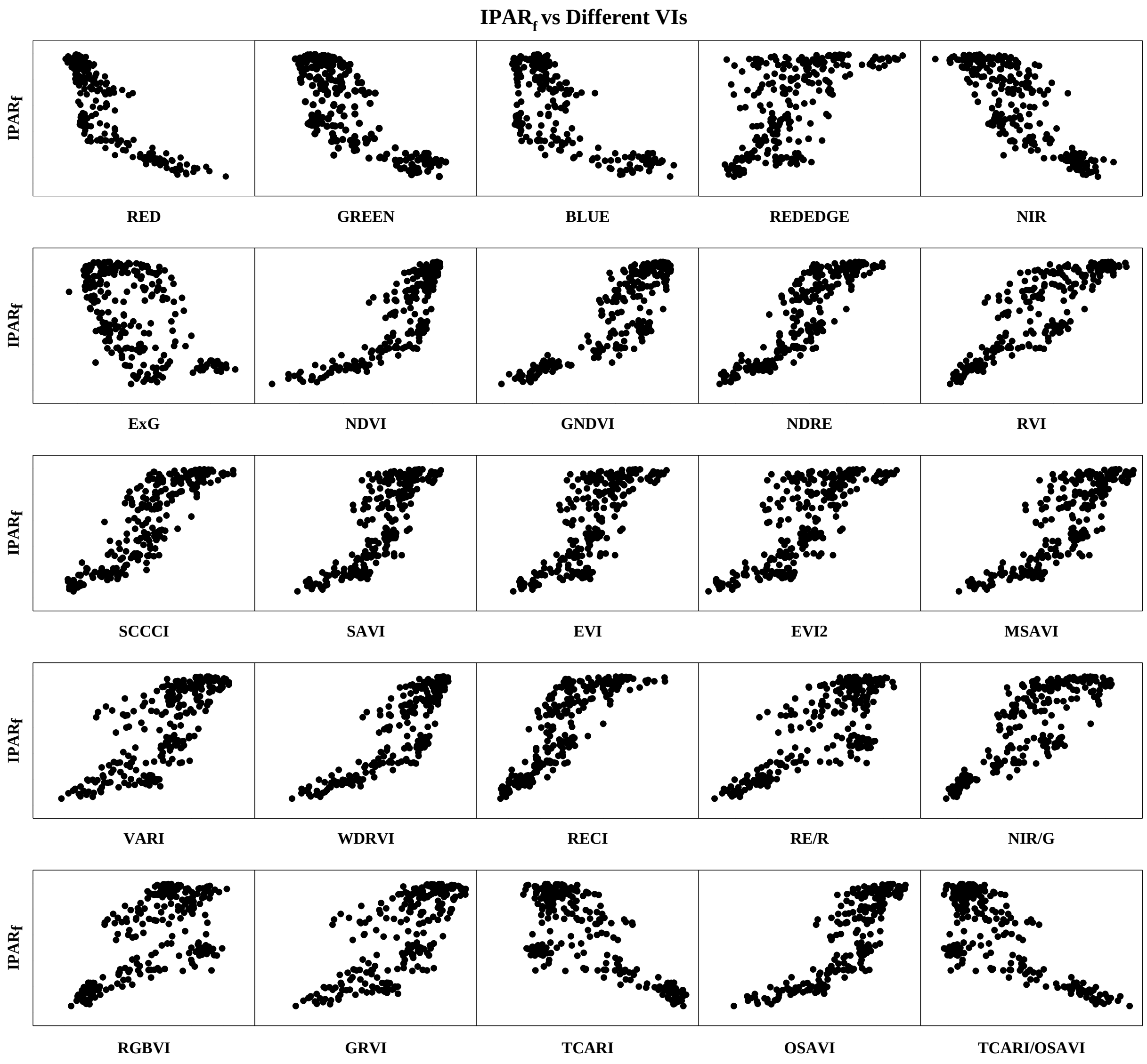

Supplementary Figure 2. Scatter Plots for fraction of Intercepted Photosynthetically Active radiation ($IPAR_f$) versus different vegetation indices (VIs) and multispectral raw bands for all data combined.

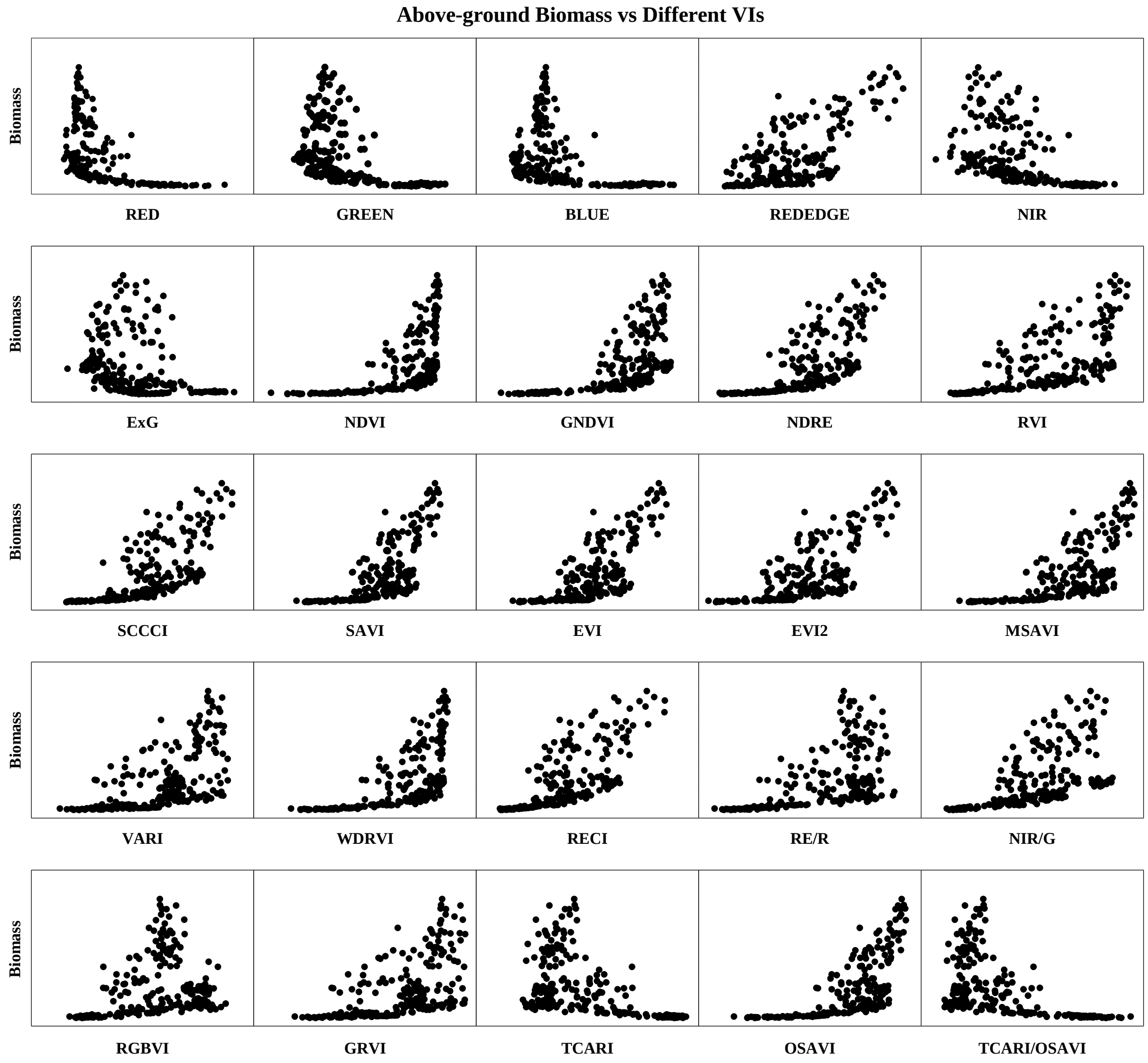

Supplementary Figure 3. Scatter Plots for above-ground biomass vs different vegetation indices (VIs) and multispectral raw bands for all data combined.

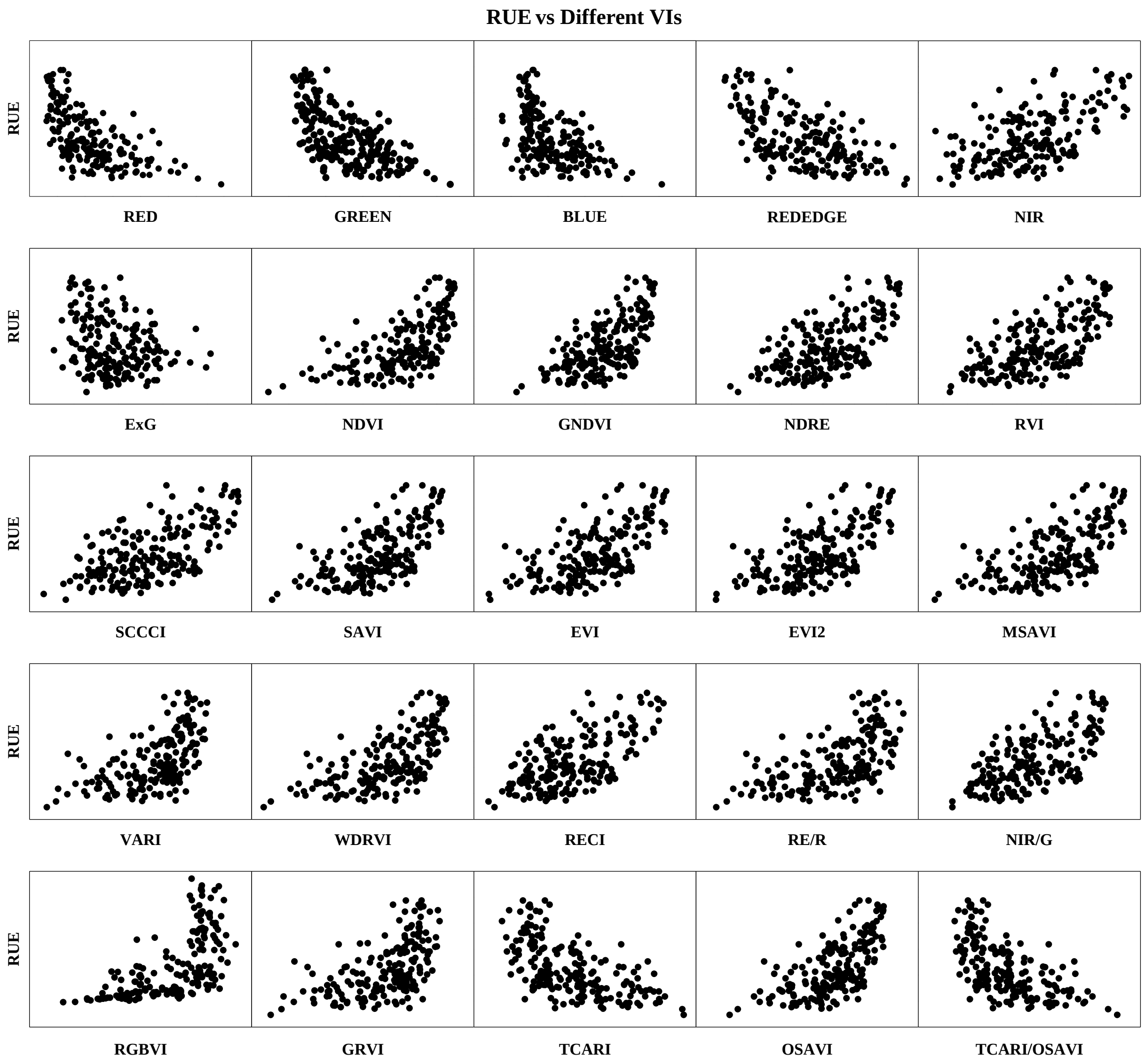

Supplementary Figure 4. Scatter Plots for radiation use efficiency (RUE) versus different vegetation indices (VIs) and multispectral raw bands for all data combined.

1.2 Supplementary Tables

Supplementary Table 1. Model equations for predicting fraction of Intercepted Photosynthetically Active Radiation ($IPAR_f$) using different vegetation indices (VIs) and raw bands in integration with GDD for the 2021 and 2022 growing seasons.

VIs	Model Equations
RVI	1 / (1 + Exp (-((-4.009) + 0.00268 * GDD + 0.182 *RVI)))
RECI	1 / (1 + Exp (-((-3.964) + 0.00241 * GDD + 1.917 * RECI)))
NDRE	1 / (1 + Exp (-((-5.214) + 0.00228 * GDD + 10.081 * NDRE)))
SCCCI	1 / (1 + Exp (-((-6.749) + 0.00229 * GDD + 11.818 * SCCCI)))
WDRVI	1 / (1 + Exp (-((-3.217) + 0.00252 * GDD + 4.376 * WDRVI)))
MSAVI	1 / (1 + Exp (-((-7.266) + 0.00277 * GDD + 8.076 * MSAVI)))
NIR/G	1 / (1 + Exp (-((-4.564) + 0.00271 * GDD + 0.376 *NIR/G)))
OSAVI	1 / (1 + Exp (-((-9.581) + 0.00262 * GDD + 10.809 * OSAVI)))
SAVI	1 / (1 + Exp (-((-8.313) + 0.00284 * GDD + 10.251 * SAVI)))
VARI	1 / (1 + Exp (-((-3.875) + 0.00304 * GDD + 6.458 * VARI)))
NDVI	1 / (1 + Exp (-((-10.194) + 10.438 * NDVI + 0.00255 * GDD)))
RE/R	1 / (1 + Exp (-((-5.108) + 0.00305 * GDD + 0.600 *RE/R)))
EVI2	1 / (1 + Exp (-((-8.148) + 0.00306 * GDD + 8.519 * EVI2)))
EVI	1 / (1 + Exp (-((-7.765) + 0.00301 * GDD + 7.771 * EVI)))
GRVI	1 / (1 + Exp (-((-3.911) + 0.00317 * GDD + 8.373 * GRVI)))
REDEDGE	1 / (1 + Exp (-(3.165 + 0.00260 * GDD - 21.843 * REDEDGE)))
GNDVI	1 / (1 + Exp (-((-9.671) + 0.00250 * GDD + 10.870 * GNDVI)))
RED	1 / (1 + Exp (-(0.189 + 0.00257 * GDD - 38.587 * RED)))
RGBVI	1 / (1 + Exp (-((-4.255) + 0.00360 * GDD + 0.0496 * RGBVI)))
TCARI	1 / (1 + Exp (-((-1.625) + 0.00310 * GDD - 6.282 * TCARI)))
TCARI/OSAVI	1 / (1 + Exp (-((-1.517) + 0.00299 * GDD - 3.931 * TCARI/OSAVI)))
GREEN	1 / (1 + Exp (-(0.524 + 0.00285 * GDD - 33.282 * GREEN)))
BLUE	1 / (1 + Exp (-((-0.344) + 0.00325 * GDD - 62.650 * BLUE)))
NIR	1 / (1 + Exp (-((-6.374) + 0.00363 * GDD + 8.231 * NIR)))
ExG	1 / (1 + Exp (-((-1.013) + 0.00368 * GDD - 21.460 * ExG)))

Supplementary Table 2. Model equations for predicting above-ground biomass using different vegetation indices (VIs) and raw bands in integration with GDD for the 2021 and 2022 growing seasons.

VIs	Model Equations
MSAVI	Exp (0.494 + 0.00238 * GDD + 4.943 * MSAVI)
OSAVI	Exp ((-1.495) + 0.00234 * GDD + 7.322 * OSAVI)
RVI	Exp (2.695 + 0.00256 * GDD + 0.0817 * RVI)
SAVI	Exp (0.095 + 0.00232 * GDD + 6.050 * SAVI)
EVI	Exp (0.859 + 0.00234 * GDD + 4.116 * EVI)
EVI2	Exp (0.606 + 0.00236 * GDD + 4.587 * EVI2)
WDRVI	Exp (2.749 + 0.00246 * GDD + 2.765 * WDRVI)
NIR	Exp (1.489 + 0.00253 * GDD + 4.791 * NIR)
NDVI	Exp ((-2.345) + 0.00242 * GDD + 7.483 * NDVI)
NDRE	Exp (2.157 + 0.00234 * GDD + 4.662 * NDRE)
VARI	Exp (2.942 + 0.00267 * GDD + 2.622 * VARI)
RECI	Exp (3.027 + 0.00241 * GDD + 0.667 * RECI)
GRVI	Exp (2.906 + 0.00271 * GDD + 3.564 * GRVI)
SCCCI	Exp (1.607 + 0.00231 * GDD + 5.219 * SCCCI)
NIR/G	Exp (2.099 + 0.00253 * GDD + 0.216 * NIR/G)
RE/R	Exp (1.884 + 0.00268 * GDD + 0.345 * RE/R)
RED	Exp (4.971 + 0.00246 * GDD - 24.382 * RED)
GNDVI	Exp ((-2.599) + 0.00232 * GDD + 8.676 * GNDVI)
TCARI	Exp (3.999 + 0.00262 * GDD - 4.489 * TCARI)
TCARI/OSAVI	Exp (4.090 + 0.00254 * GDD - 3.022 * TCARI/OSAVI)
RGBVI	Exp (2.640 + 0.00289 * GDD + 0.0254 * RGBVI)
BLUE	Exp (5.065 + 0.00265 * GDD - 46.343 * BLUE)
REDEDGE	Exp (5.541 + 0.00248 * GDD - 7.436 * REDEDGE)
GREEN	Exp (5.338 + 0.00236 * GDD -19.060 * GREEN)
ExG	Exp (4.091 + 0.00266 * GDD - 3.202 * ExG)

Supplementary Table 3. Model equations for predicting radiation use efficiency (RUE) using different vegetation indices (VIs) and raw bands for the 2021 and 2022 growing seasons.

VIs	Model Equations
RECI	(-0.0938) + 1.036 * RECI
NIR/G	(-0.634) + 0.249 * NIR/G
NDRE	(-0.871) + 5.636 * NDRE
SCCCI	(-1.682) + 6.498 * SCCCI
REDEDGE	4.703 -15.600 * REDEDGE
GNDVI	(-5.059) + 8.519 * GNDVI
RVI	(-0.115) + 0.107 * RVI
GREEN	3.771 -34.743 * GREEN
WDRVI	0.240 + 2.728 * WDRVI
OSAVI	(-3.735) + 6.793 * OSAVI
EVI	(-2.953) + 5.545 * EVI
NDVI	(-4.123) + 6.536 * NDVI
RED	2.533 -27.006 * RED
SAVI	(-3.256) + 7.064 * SAVI
MSAVI	(-2.355) + 5.263 * MSAVI
EVI2	(-3.547) + 6.554 * EVI2
NIR	(-3.764) + 10.202 * NIR
TCARI/OSAVI	1.606 -3.525 * TCARI/OSAVI
VARI	(-0.0034) + 4.014 * VARI
TCARI	1.604 -5.903 * TCARI
RE/R	(-1.042) + 0.432 * RE/R
GRVI	(-0.0045) + 5.337 * GRVI
BLUE	3.426 -74.987 * BLUE
RGBVI	(-0.46) + 0.0443 * RGBVI
ExG	2.008 -12.434 * ExG
